# Ideologically biased evaluation of evidence - A signal-detection approach to measure individual differences

**DOI:** 10.1371/journal.pone.0357011

**Published:** 2026-09-11

**Authors:** Arne Stolp, Christine Finn, Carolin-Theresa Ziemer, Christian Thiel, Tobias Rothmund

**Affiliations:** 1 Institute of Communication Science, Department of Behavioral and Social Science, Friedrich Schiller University of Jena, Germany; 2 Max-Planck-Institute for Human Development, Berlin, Germany; 3 Institute for Data Science, German Aerospace Center, Germany; University of Stavanger, NORWAY

## Abstract

In a context of increasing political polarization, ideological bias poses a challenge to evidence-based reasoning and the evaluation of factual information. However, its systematic study is hindered by the lack of reliable and valid measurement tools and by the frequent conflation of ideological bias with ideological stance. Addressing this gap, we introduce the Ideological Bias Expression (IBE) scale, a novel measure grounded in signal detection theory that disentangles discrimination accuracy from ideological bias in individuals’ evaluations of evidence. Across two studies using German samples (*N*_*Study1*_ = 446; *N*_*Study2*_ = 1,359), we provide initial validation of the IBE scale across two distinct political issue domains (climate change/energy & domestic security/migration). Results indicate that the IBE scale is an effective tool for assessing individual differences in biased evidence evaluation, providing a foundation for future research on its determinants and potential interventions to promote more evidence-based public discourse. Using the scale, we find evidence that ideological bias is empirically distinct from knowledge-related factors, including formal education and political knowledge, indicating that such bias cannot be reduced to deficits in knowledge or reasoning alone. Moderate coherence across two issue domains also suggests that biased evidence evaluation is not purely issue-specific. Finally, in the German political context, ideological bias appears to reflect a combination of social identity dynamics and value-based cognition rather than partisan alignment alone.

## Introduction

Political polarization has become a pressing challenge for democratic societies worldwide and political debates in particular. Polarized political debates often involve ideological conflicts over scientific evidence [1–3]. Prominent examples include debates about climate change, domestic security, or the COVID-19 pandemic, where acceptance or rejection of factual evidence often reflects ideological beliefs or political identities rather than the strength of the evidence itself [4–6]. This erosion of shared factual ground is likely to increase susceptibility to misinformation [7,8] and hamper rational public deliberation and evidence-based policymaking [9].

An important psychological driver of polarization is ideological bias, the individual tendency to selectively accept or reject scientific evidence depending on its alignment with ideological beliefs or political affiliation (e.g., [10–13]). Despite extensive evidence for the prevalence of ideological bias, there is currently no well-established measurement approach that captures individual differences in ideologically biased evaluations of evidence.

Systematic reviews in the field have pointed out that frequently used measures typically involve two methodological shortcomings. One is that ad-hoc measures are used for which reliability and validity have not been demonstrated (e.g., [14]). The other is that measures are often conflated with ideological stances as noted in research on conspiracy beliefs [15]. Both are problematic, given that reliable and valid measures are needed to test the effectiveness of interventions to reduce ideological bias.

Addressing these shortcomings, the main goal of this research is to introduce and operationalize a measurement approach based on signal detection theory [16] that allows the reliable and valid assessment of interindividual differences in ideological bias. A second goal is to utilize the measure to test three disputed theoretical assumptions (RQ1–3) discussed in the empirical literature, concerning (a) the relation of ideological bias and knowledge, (b) the coherence of ideological bias across two political issues, and (c) ideological identity- and value-related aspects of bias.

### Ideologically biased evaluation of evidence

Numerous studies have shown that political ideology shapes how individuals perceive, interpret, and evaluate factual and scientific evidence (e.g., [6,11,17–19]). For example, Washburn and Skitka [13] demonstrated that both left-wing and right-wing individuals interpreted the same scientific data differently, depending on whether or not the results aligned with their political beliefs. This can involve two complementary modes of biased processing: confirmation bias and disconfirmation bias [20]. Confirmation bias describes the tendency to uncritically accept evidence that is congruent with a person’s political worldview [21], which can lead individuals to accept weak or false evidence [22]. Disconfirmation bias reflects the tendency to overcritically scrutinize and dismiss evidence that is incongruent with a person’s political worldview, which can lead individuals to neglect strong scientific evidence [22].

Different labels have been used to describe this ideologically biased evaluation of evidence, such as partisan bias [23], myside-bias [24] or political bias [20]. We suggest using the term ideological bias as an umbrella concept describing a one-sided evaluation of factual evidence based on ideological alignments. These alignments have been linked to cognitive and motivational underpinnings. Drawing on dual-process theories of cognitive reasoning (for an overview, see Gawronski & Creighton [25]), some scholars argue that ideological bias may result from heuristic information processing (e.g., [26]). When individuals are strongly engaged in ideological alignments, they may rely on a consistency heuristic, treating the congruency between new information and their existing worldview as a heuristic cue for credibility when evaluating new evidence [27]. Based on this reasoning, cognitive accounts suggest that such biased evaluation of evidence is mainly the result of insufficient cognitive reflection or limited knowledge [28]. However, motivational accounts challenge this assumption by suggesting that ideological bias results from the motivation to defend political worldviews (e.g., [29–31]). For example, Kahan et al. [11] demonstrated that individuals with higher numeracy and scientific literacy often use these skills selectively to defend prior beliefs rather than to revise them. Similarly, Stanovich et al. [24] argue that biased reasoning operates independently of general intelligence. In this line of thinking, cognitive reflection and political knowledge are of instrumental value to inform a reasoning process that is directed towards reaching a desired conclusion.

Consequently, different theoretical accounts expect different relations between the strength of ideological bias and the amount of cognitive reflection and political knowledge: Cognitive accounts expect a negative relation (e.g., [28]), motivational accounts expect a positive relation [32]. Clarifying this contradiction is important, given that different approaches for interventions follow from each assumption. These range from accuracy nudges, intended to enhance analytical thinking and knowledge under cognitive accounts (e.g., [33]), to targeting identity-focused interventions under motivational accounts (e.g., [34]). Based on these considerations, the current work aims to clarify the relation between ideological bias expression and indicators for political knowledge.

Another theoretical debate related to ideological bias concerns the construct validity of political ideology as a psychological concept: How ideologically coherent is political thinking in laypersons? Political scientists have been concerned with this question since the seminal publications of Lane [35] and Converse [36]. Some scholars assume that citizens are “innocent” of ideology in the sense that they do not engage in ideologically coherent ways of thinking about politics. For example, Kinder and Kalmoe [37] argue that, “People are busy with more pressing things; politics is complicated and far away. Ideology is not for them.” If this was true, ideological bias should be relatively inconsistent across political topics or domains of scientific evidence. For example, people who are ideologically engaged and biased in the evaluation of evidence supporting a liberal political project (e.g., expansion of renewable energy) are not more likely to be biased about evidence supporting another liberal project (e.g., providing individual rights for political asylum). In fact, if this was true, the term ideological bias might be misleading, given that biased evaluation of evidence would then be issue-specific, for example, as an ideological bias specific to climate change. Although some studies focus on issue-specific biases (e.g., climate change: [38]; COVID-19: [39]), other scholars have opposed this view and argued that there is a substantial amount of ideological congruence in political thinking even among laypersons (e.g., [40,41]). This assumption suggests that there is also some coherence between ideological bias across ideologically related issues, pointing to a broader phenomenon that is not tied to a specific context (e.g., [42,43]). We aim to investigate the empirical coherence of ideological bias expression across ideologically related topics in the second research question.

In the political domain, two sources for ideological alignments are discussed: (a) party identification [44] and (b) political values [45]. When political party affiliation constitutes a salient social identity, individuals may engage in partisan bias, evaluating evidence in ways that align with their party’s agenda and identity [46]. However, ideological bias may also stem from deeply held political values, such as authority, equality, fairness, or liberty, that shape broader worldviews [43] and stances on more specific issues [45]. Importantly, much of the empirical literature on ideologically biased evaluation of evidence has been conducted in two-party systems such as the United States, where party identification and political value orientation are highly correlated. In such contexts, it is empirically difficult to separate whether biased evaluations primarily reflect the protection of group identity or the defense of political values. In multi-party systems, however, party identification and ideological beliefs are less tightly coupled. Individuals may share similar ideological values while supporting different parties or may hold strong ideological commitments without clear partisan affiliation. Although some studies demonstrate that political or moral values predict worldview defense independently of party affiliation [46–48], it remains an open question whether ideological bias is independently driven by partisan identity or by political value commitments. To address this issue, we aim to examine the unique contributions of both factors in predicting ideological bias in a multi-party context, where party affiliation and ideological values are less tightly aligned.

Despite extensive evidence for ideologically biased evaluation of evidence, most empirical approaches rely on group-level differences in experimental designs. Typically, bias is inferred from differences in how participants evaluate identical information when it is framed as ideologically congruent versus incongruent (e.g., [18,49,50]). While this paradigm has been effective in demonstrating the existence of motivated reasoning, it primarily captures average effects at the group level and does not allow for a direct assessment of interindividual differences. As a result, it remains unclear whether ideological bias reflects a generalizable individual tendency or context-specific responses to particular stimuli. This limitation is particularly relevant for the present research questions, which concern both the relationship between ideological bias and individual characteristics, and its coherence across domains. Addressing these questions requires a measurement approach that captures individual variability. Recent research on misinformation susceptibility (e.g., [51,52]) suggests signal detection theory [16] as a formal framework to provide tools to separate sensitivity to factual correctness from systematic bias at the individual level. Building on this approach, the present research conceptualizes ideological bias as a psychological construct that captures individual differences in the evaluation of evidence across contexts.

### IBE measurement concept

Our measurement approach builds on and extends the empirical finding that ideological bias reflects a one-sided evaluation of evidence based on ideological alignments. More specifically, it can be expected that people tend to accept ideologically congruent claims as true (confirmation bias) and reject ideologically incongruent claims as false (disconfirmation bias; [20]). For example, people who strongly support climate protection as a political value or identify with a green political party are more likely to believe empirical claims that speak to the urgency of political action compared to people who believe that climate change as a political topic is overrated.

The goal of our measure is to capture interindividual differences in ideological bias by calculating difference scores for performance across two tasks, Task-Left and Task-Right. Both tasks involve the truth evaluation of a series of empirical claims about an ideologically polarized issue. In other words, our measure is implemented as a decision task in which participants evaluate evidence-based statements by judging whether a claim is true or false. In both tasks, the correct response option can either require respondents to accept a claim as true or to reject a claim as false. Importantly, in Task-Left, the correct response is always congruent with a left-wing political position. In Task-Right, the correct response is always congruent with a right-wing political position. Crossing factual correctness with ideological congruence yields four distinct item types. Different examples for claims in Task-Left and Task-Right are outlined in Table 1.

**Table 1:** Item Categories with Exemplary Items from the Issue-Domain Climate Change.

Truth Value of Claim	Ideological Congruence of Correct Response	
	Task-Left	Task-Right
True	The summer ice-covered surface of the Arctic was about one-third larger in 1980, compared to today’s.^1^	A vehicle with an electric drive requires more energy to produce than a vehicle with an internal combustion engine.^2^
False	Per capita CO2 emissions in Germany are significantly lower than in China.^3^	Greenhouse gas emissions in Germany are still increasing every year.^4^

*Note.* ^*1*^ *Alfred Wegener Institute & University of Bremen (2020): Sea Ice.de – Monthly means of sea ice extent;* ^*2*^ *General German Automobile Association (2022): Life Cycle Analysis Tool;* ^*3*^ *IEA (2024): Global Carbon Atlas 2024;* ^*4*^ *Federal Environment Agency (2024): Greenhouse gas emissions in Germany since 1990 by Gases.*

Based on this empirical framework, the central idea of our measure is to quantify a within-person asymmetry in evaluation accuracy compared between Task-Left and Task-Right. Unbiased evaluation would be reflected in comparable performance across both tasks. In contrast, ideological bias manifests as a systematic tendency to evaluate claims more accurately when the correct response is congruent with ideological alignments. A stronger performance asymmetry favoring Task-Left indicates a higher left-wing ideological bias, whereas a stronger asymmetry favoring Task-Right indicates a higher right-wing ideological bias. In other words, this measurement logic quantifies how accurately an individual tends to evaluate the truth value of evidence based on whether their response supports or challenges their ideological predispositions. This measurement not only allows us to quantify ideological bias but also enables a systematic distinction between left-wing and right-wing political bias.

### The present research

The present research pursues two goals. First, we aim to develop a reliable and valid scale to assess interindividual differences in ideological bias. In Study 1, we report the operationalization process and the assessment of indicators of item and scale quality. In Study 2, we use this measure to investigate three highly disputed research questions concerning ideological bias in a German political context.

We examine whether ideological bias reflects deficits in knowledge, as suggested by heuristic-processing accounts (e.g., [28]). Contradicting this claim, theoretical and empirical work on motivated reasoning indicate that individuals with higher scientific literacy often use these skills selectively to defend prior beliefs rather than revise them [11,47,50].

*RQ1: What is the relation between ideological bias expression and political knowledge?*

Assuming ideological alignment systematically shapes evidence evaluation, we test whether bias expression is related across two assessed domains [41]. Although some studies focus on issue-specific biases (e.g., climate change: [38]; COVID-19: [39]), accumulating evidence suggests an overarching phenomenon that is not tied to specific areas (e.g., [42,43,53]).

*RQ2: How coherent is ideological bias expression across ideologically related issue domains?*

Drawing on prior evidence that both partisan identity [46] and ideological values [45] contribute to biased reasoning it remains an open question whether ideological bias is independently driven by partisan identity and by ideological value commitments, especially in a multi-party context, where party affiliation and ideological values are less tightly aligned.

*RQ3. Do party identification and political values independently predict ideological bias expression?*

### Study 1 – IBE Scale Development

In Study 1, we developed, operationalized and evaluated our measure of individual differences in ideological bias expression. We drew on two political issues as domains of high political polarization, namely (a) climate change/renewable energy and (b) migration/domestic security. These issue domains were selected because they provide clear contrasts in policy-related ideological positions in the German political debate. In the climate domain, left-leaning positions typically emphasize the risks of climate change and the urgency of decarbonization, whereas right-leaning positions often highlight economic costs and express skepticism toward renewable energy transitions [54,55]. In the domestic security domain, right-leaning positions tend to emphasize risks associated with immigration, while left-leaning positions more often stress stable or declining overall crime rates [56,57].

In the following section we describe item development and then introduce the signal detection framework [16], an analytical procedure used to calculate two parameters: (i) a standard SDT indicator of task performance, and (ii) a new indicator of ideological bias expression. We summarize item selection in a German sample (*N*_*Study1*_ = 446) and report detailed item analyses and scale assessments.

### Item Construction

We started the process of item development by investigating empirical or scientific evidence in both political issue domains to derive well documented, narrowly defined, descriptive facts from publicly available datasets. For example, facts were derived from scientific and official statistical sources, including the IPCC Sixth Assessment Report [58] or the Polizeiliche Kriminalstatistik 2023 V6.0 [59].

Based on these facts, we generated claims that met three criteria: First, each claim was clearly true or false based on the underlying evidence. To reduce ambiguity, we avoided evaluative or causal language in each claim and focused on concrete descriptive statements. Second, each claim needed to be well comprehensible. Although many of these facts involve complex phenomena, we aimed for our measure to be valid across varying educational levels. To achieve this, we conducted pretests to optimize item wording and ensure clarity through simple language in order to reduce the influence of language comprehension on evaluation accuracy across claims of varying difficulty. Third, the correct response to each claim (which could be true or false) was either in line with a left-leaning or a right-leaning ideological position on the given issue. Importantly, this alignment focuses on issue-specific policy positions prevalent in contemporary German political discourse. For example, the statement “Greenhouse gas emissions in Germany are still increasing every year” is false [60]; correctly rejecting this claim reflects a less climate-concerned position, more commonly associated with right-leaning political actors. Again, we conducted pretests to optimize this criterion and investigated whether individuals who reported left-wing or right-wing ideological identity were more likely to select the respective response option. Finally, a list of 30 candidate items for the two political issue domains was constructed to cover all four combinations of factual correctness and ideological congruence. In S2 Table (see Supporting Information) each claim is documented with the source of its underlying empirical basis and a reference that reflects a typical issue-specific political framing in contemporary German discourse, in line with the claim’s correct evaluation.

### Scale Indices

At a conceptual level, the measurement approach is grounded in a signal detection theory framework (SDT; [16]) and distinguishes between two key indices: (i) discrimination sensitivity is the established SDT parameter *d’* indicating overall task performance in terms of accuracy. (ii) ideological bias expression *b’* is an SDT-inspired indicator that quantifies systematic asymmetries in discrimination sensitivity across ideologically congruent versus incongruent tasks. As such, *b′* is not an SDT response-criterion parameter (e.g., *c* or *β*) but was developed to capture performance differences across item sets. Both indices can be computed separately for each issue domain as well as across domains.

Discrimination sensitivity (*d′*) reflects a respondent’s ability to distinguish true from false claims. It is calculated as the difference between the standardized hit rate, i.e., the proportion of true claims correctly accepted (Hits), and the standardized false alarm rate, i.e., the proportion of false claims incorrectly accepted (False Alarms). Higher *d′* values indicate greater discrimination accuracy, whereas a value of zero reflects chance-level performance.


$$\begin{array}{c} d^{\prime }\;=\;z(hit\;rate)\;-\;z(false\;alarm\;rate) \end{array}$$

SDT-typical classification of responses used to derive *d′* are illustrated below (Table 2).

**Table 2:** *d’* calculation categories.

Truth Value of Claim	Response		Total
	„True“	„False“	
True	H	M	Signal Trial
False	FA	CR	Noise Trial
Total	Acceptance Responses	Rejection Responses	

*Note. H = Hit; M = Miss; FA = False Alarm; CR = Correct Rejection.*

In addition to discrimination sensitivity, we propose an index for ideological bias expression (*b′*) that captures asymmetries in discrimination sensitivity across two tasks that differ in the ideological alignment of the correct response. For its calculation, response data are reorganized into a 2 × 2 matrix defined by the ideological congruence of the correct response (Task-Left vs. Task-Right) and response correctness (accurate vs. inaccurate; see Table 3). Based on this structure, *b′* is computed as the difference between the standardized correct response rate, i.e., the proportion of correct responses (both Hits and Correct Rejections) for Task-Right and the standardized correct response rate for Task-Left.

**Table 3:** *b’* calculation categories.

Congruence of Correct Response	Response		Total
	Accurate	Inaccurate	
Task-Right	H + CR	M + FA	Right Congruent Trials
Task-Left	H + CR	M + FA	Left Congruent Trials
Total	Correct Responses	Incorrect Responses	

*Note. H = Hit; M = Miss; FA = False Alarm; CR = Correct Rejection.*


$$\begin{array}{c} b^{\prime }\;=\;z(Task-Right\;correct\;rate)\;-\;z(Task-Left\;correct\;rate) \end{array}$$

Positive values indicate relatively higher accuracy within Task-Right, corresponding to a right-leaning bias, negative values indicate higher accuracy within Task-Left, corresponding to a left-leaning bias. Values around zero indicate no systematic asymmetry. Importantly, unlike the response criterion, *b′* does not capture a general tendency to respond “true” or “false,” but instead reflects intraindividual asymmetries in evaluative performance as a function of ideological congruence.

In the following, we present an exemplary calculation for two participants showing the same discrimination sensitivity but differences in ideological bias expression. Participant 1 performed equally well in Task-Left and Task-Right. This symmetrical performance across tasks indicates no ideological bias expression (*b’* = 0) and a reasonably high discrimination sensitivity (*d’* = 1.05). Participant 2 has a positive value for *b’* (corresponding to a right-leaning bias) due to higher discriminative accuracy in Task-Right, where the correct response aligns with right-congruent positions, compared to Task-Left, where the correct response aligns with left-congruent positions.

**Box 1. Example for Discrepancies between Discrimination Sensitivity (*d’*) and Ideological Bias Expression (*b’*).**

**Table:** 

Participant 1 No Bias				Participant 2 Right-leaning Bias			
Item Type	Response		Total	Item Type	Response		Total
	Accurate	Inaccurate			Accurate	Inaccurate	
Task-Right	7	3	10	Task-Right	9	1	10
Task-Left	7	3	10	Task-Left	5	5	10
Total	14	6		Total	14	6	
Calculation of overall and domain-specific discrimination sensitivity (identical for both participants): $d^{\prime }\;=\;z(\text{0}.\text{7}\text{0})\;-\;z(\text{0}.\text{3}\text{0})\;=\;\text{0}.\text{5}\text{2}\text{4}\;-\;(-\text{0}.\text{5}\text{2}\text{4})\;=\;\text{1}.\text{0}\text{4}$							
Calculation of *b’* for participant 1: $b^{\prime }=z(\text{0}.\text{7}\text{0})-z(\text{0}.\text{7}\text{0})=\text{0}.\text{5}\text{2}-\text{0}.\text{5}\text{2}=\text{0}$				Calculation of *b’* for participant 2: $b^{\prime }=z(\text{0}.\text{9}\text{0})-z(\text{0}.\text{5}\text{0})=\text{1}.\text{2}\text{8}-\text{0}.\text{0}\text{0}=\text{1}.\text{2}\text{8}$

## Methods

### Participants

Participants were recruited via a German panel provider (Cint Deutschland GmbH) using quota sampling to approximate population distributions in education, voting behavior, age, and gender. Panelists received monetary compensation for their participation. Data collection took place in July 2024. Of 1,409 panelists who accessed the survey, exclusions were due to non-start (17), dropout (16), failed attention checks (25), ineligibility (1) or fulfilled quotas (894). Detailed quota distributions and population benchmarks are reported in S1A and B Table in the Supporting Information. After additionally removing speeders (top 2% fastest completion times), the final analytic sample comprised *N*_*Study1*_ = 446 participants (*M*_*age*_ = 50.47, *SD*_*age*_ = 14.5, 50.45% female). Median completion time was 12.12 minutes.

### Procedure

Participants first provided informed consent, including the voluntary nature of participation, their right to withdraw at any time without penalty, and the anonymous handling of their data. Following consent, they completed demographic questions (age, gender, education, and voting behavior) and the 30-item randomized IBE task. Participants then completed the measures of political knowledge and political orientation described below. Upon completion of the study, participants were debriefed about the purpose of the research.

### Measures

**Ideological Bias.** The IBE scale used in Study 1 comprised the 30 candidate items resulting from the item construction procedure described above (see Item Construction). These statements covered two political domains, migration/domestic security and climate change/renewable energy, and were systematically balanced across factual correctness (correct vs. incorrect) and the ideological congruence of the correct response (Task-Left vs. Task-Right). Participants evaluated each statement in a dichotomous format (true vs. false).

**Political Orientation.** Political orientation was measured using an 11-point scale ranging from 1 (left) to 11 (right), a well-established and widely used operationalization consistent with Kroh [61] that has strong predictive validity for political attitudes and behavior.

**Political Knowledge.** The Hohenheimer Inventory of Political Knowledge (HIP; [62]) assesses different dimensions of political knowledge, a) Fundamentals (state structures and historical facts) and b) Current Affairs (topics and actors across various political fields), validated in a German population. We implemented 10 items from the subscale b) Current Affairs which primarily capture domain-specific political knowledge, rather than general civic knowledge and therefore reflect participants’ familiarity with contemporary political issues and actors. For example, the scale included the item *„What does the IPCC institution deal with?“.* For each item, a single-choice response format with four answer options was used. The exemplary item included the following response options

a) Drug misuse b) Climate change c) Migration d) None of the other answer options are correct, where b) denoted the correct option. The scale indicator was calculated as the sum of the correct responses and showed acceptable internal consistency (*α* = .67).

## Results

### Item Selection

Item difficulty was first calculated for all candidate items, followed by logistic regression analyses predicting correct responses from political knowledge and political orientation. Items were retained based on four criteria: (a) both predictors significantly influenced accuracy in the theoretically expected direction, (b) item difficulty ranged between 10% and 90% (i.e., proportion of correct responses), (c) redundancy was minimized by excluding items referring to the same or highly similar facts, and (d) the final selection ensured a heterogeneous distribution across the four response categories (see Table 1).

Applying these criteria resulted in the exclusion of (a) twelve items due to non-significant or theoretically inconsistent predictor effects, and (b) three additional items due to exceeding the predefined difficulty threshold. Two items outside the difficulty range were nevertheless retained to preserve balanced category distributions. The final set comprised 20 items, for which *d′* and *b′* were computed both by domain and across all items, alongside item-scale correlations to assess discrimination. A complete overview of all candidate items and the results of the logistic regression analyses are provided in S4 Table in the Supporting Information.

### Scale Intercorrelations

The overall and domain-specific discrimination sensitivity (*d’*) and ideological bias expression (*b’*) were calculated based on the final set of 20 items. Correlations among IBE indices are reported in Table 4, indicating weak negative associations between estimates for ideological bias expression and discrimination sensitivity for the overall measure (*r* = −.16, *p* = .001) as well as within the issue of climate change/renewable energy (*r* = −.23, *p* < .001) while no association occurred for the issue of domestic security (*r* = .01, *p* = .885). Similarly, slight negative relationships between *b’* and political knowledge (HIP, *r*_*M*_ = −.14, *p* = .011; *r*_*CC*_ = −.16, *p* < .001; *r*_*DS*_ = −.06, *p* = .54) and education level (*r* = −.17, *p* < .001) indicate that higher general political knowledge was only weakly associated with a more left-leaning bias. No association was found with security related claims. The issue-specific bias estimates showed a positive and significant intercorrelation (*r* = .35, *p* < .001) and were strongly associated with the overall bias mean (*rs* = .75 to.77, *ps* = < .001).

**Table 4:** Study 1: Intercorrelations Among Estimates for Ideological Bias Expression and Discrimination Sensitivity.

Variables	*M (SD)*	*b’*_*CC*_	*b’*_*DS*_	*d’*_*M*_	*d’*_*CC*_	*d’*_*DS*_
*b’*_*M*_	.19 (.76)	.80	.84	−.16	−.16	−.08
*b’*_*CC*_	−.22 (.88)		.35	−.25	−.23	−.15
*b’*_*DS*_	.61 (.96)			−.04	−.04	.01
*d’*_*M*_	.55 (.54)				.77	.75
*d’*_*CC*_	.33 (.72)					.14
*d’*_*DS*_	.76 (.7)					

*Note. N*_*Study1*_ *= 446; b’ = ideological bias expression; d’ = discrimination sensitivity; CC = issue domain climate change/renewable energy; DS = issue domain domestic security/migration.*

### Item characteristics and item-scale correlations

The mean item probability of a correct response (*P*), indicating item difficulty, and item-scale correlations (*r(d’)*), interpreted in terms of item discriminability are reported in Table 5. As expected, in the climate change/renewable energy domain, items from Task-Left (items 1–6) correlated negatively with overall *b’* (*r* = −.21 to −.40), indicating that correct responses on these items contributed to a more left-leaning bias. By contrast, items from Task-Right (items 11–14) showed positive correlations with bias scores (*r* = .30 to.48), consistent with a right-leaning contribution. A similar pattern emerged in the domestic security/migration domain. Items from Task-Left (items 7–10) correlated negatively with bias (*r* = −.44 to −.47), whereas items from Task-Right (items 15–20) correlated positively (*r* = .09 to.35).

**Table 5:** Item-Scale Correlations of Final Item-Set.

No.	Ideol. Congruence of Correct Response	Issue Domain	*P*	*r*(*d’*)			*r*(*b’*)		
				*d’*_*M*_	*d’*_CC_	*d’*_DS_	*b’*_*M*_	*b’*_CC_	*b’*_DS_
1	Task-Left	CC	.51	.36	.46	.07	−.40	−.54	−.14
3	Task-Left	CC	.89	.24	.21	.15	−.16	−.25	−.03
4	Task-Left	CC	.60	.39	.51	.08	−.26	−.37	−.07
5	Task-Left	CC	.73	.26	.27	.12	−.32	−.39	−.14
6	Task-Left	CC	.26	.35	.41	.11	−.39	−.43	−.22
9	Task-Left	CC	.79	.25	.31	.07	−.20	−.26	−.08
11	Task-Left	DS	.33	.10	.02	.13	−.51	−.20	−.62
12	Task-Left	DS	.86	.20	.02	.29	−.40	−.29	−.36
13	Task-Left	DS	.48	.44	.18	.49	−.44	−.24	−.47
16	Task-Left	DS	.41	.26	.04	.36	−.48	−.21	−.56
17	Task-Right	CC	.26	.05	.15	−.07	.37	.49	.14
19	Task-Right	CC	.78	.02	.03	−.01	.50	.49	.34
20	Task-Right	CC	.35	.24	.39	−.03	.29	.39	.10
21	Task-Right	CC	.79	.17	.24	.02	.29	.35	.14
22	Task-Right	DS	.47	.22	−.01	.35	.38	.14	.47
24	Task-Right	DS	.95	.20	.08	.26	.20	.09	.20
26	Task-Right	DS	.76	.26	.04	.35	.25	.05	.34
28	Task-Right	DS	.81	.31	.06	.41	.24	.01	.37
29	Task-Right	DS	.87	.14	−.02	.24	.29	.11	.35
30	Task-Right	DS	.67	.21	.03	.29	.30	.09	.39

*Note. N*_*Study1*_ *= 446; P = mean item probability of a correct response; d′ = discrimination sensitivity; b′ = ideological bias expression; Subscripts denote issue domains (M = overall/mean scale, CC = climate change/renewable energy, DS = domestic security/migration); Positive correlations indicate greater alignment with the respective construct; negative correlations indicate inverse relationships.*

With regard to the issue-specific scores, the sample showed a higher discrimination sensitivity for the domestic security/migration issue (*d’*_DS_ = .76, *SD* = .7) compared to the climate change/renewable energy issue (*d’*_CC_ = .33, *SD* = .72). In addition, the sample showed a slight left-leaning ideological asymmetry on the climate/energy issue (*b’*_CC_ = −.22, *SD* = .88) in contrast to a more pronounced right-leaning ideological asymmetry on the security/migration issue (*b’*_DS_ = .61, *SD* = .96). The overall mean values indicated moderate discrimination sensitivity across issues (*d’*_*M*_ = .55, *SD* = .54) and a small right-leaning overall bias (*b’*_*M*_ = .19, *SD* = .76). An overview, summarizing the overall tasks’ difficulty and scale-discrimination, separately by issue domain, is provided in S6 Table in the Supporting Information.

### Reliability and Robustness of Indices

The internal consistency reliability of the task-derived indices was estimated using a fixed split-half procedure with Spearman-Brown correction, enabled by access to trial-level data [63]. To ensure comparability, item subsets were constructed to be balanced across Task-Left and Task-Right (see Table 1), mirroring the structure of the full scale. Results indicate poor reliability for *d’* (*r* = .13; *Spearman-Brown* = .23) but acceptable reliability for *b’* (*r* = .49; *Spearman-Brown* = .65). Reliability estimates for all indices across Study 1 and Study 2 are reported in S5 Table in the Supporting Information.

To address potential item-level property confounds we estimated a multilevel logistic regression model predicting item-level correctness from participants’ ideological self-placement (standardized), the ideological congruence of the correct response (Task-Left vs. Task-Right), and their interaction. Random intercepts were included for items and participants to account for differences in item difficulty and individual accuracy. The critical Ideology × Congruence interaction remained statistically significant (*b* = 0.601, *SE* = 0.051, *p* < .001), indicating that the probability of correctly evaluating a claim systematically depended on the match between participants’ ideological position and the ideological alignment of the correct response. Importantly, this effect persisted after controlling for both item-level variance and general accuracy differences between individuals. These findings suggest that the observed asymmetry in evaluation performance cannot be explained solely by differences in item difficulty or participants overall accuracy differences. To examine whether the observed interaction was driven by item-specific variation in ideology effects, we estimated an additional multilevel logistic model allowing the effect of ideology to vary across items. The Ideology × Congruence interaction remained virtually unchanged (*b* = 0.607, *SE* = 0.064, *p* < .001), indicating that the observed effect was robust to item-specific variation in ideology effects. The results of the multilevel logistic regression models are reported in S7 Table in the Supporting Information, along with an overview, reporting the tasks’ mean difficulty separately by issue domain (S6 Table).

Finally, we conducted a robustness analysis to address concerns about expressive or satisficing responding, excluding the fastest 10% of respondents who are more likely to provide rapid and potentially low-effort responses. The resulting estimates were virtually identical to those obtained in the full sample (*r* = 1.00 for both *d′* and *b′*), indicating that the observed estimates are not driven by speeding artifacts or low-effort responding.

## Discussion

The primary goal of Study 1 was to identify and select a set of items for measuring ideological bias across two politically polarized domains: climate change/renewable energy and domestic security/migration. The selection process yielded a balanced set of items that met psychometric standards regarding difficulty and item-scale discrimination. Item-level analyses, as well as multilevel logistic modeling suggest that ideological congruence consistently predicted the correctness of responses, independent of item difficulty and individual discrimination accuracy. Correct evaluations on items from Task-Left were associated with more left-leaning bias scores, whereas correct evaluations on items from Task-Right contributed to more right-leaning bias scores. These consistent directional relationships between item congruence and overall bias confirm that the selected items function as intended in capturing ideologically biased evaluation of evidence.

The weak associations between estimates of *d’* and *b’* (see Table 4) do not support a profound interdependence between the ability to discriminate the truth value of evidence and ideologically biased responding. Relationships between bias estimates and political knowledge indicators were similarly small, as was the association with educational level. These patterns suggest that higher levels of general political knowledge and education are only weakly related to a more left-leaning bias expression, particularly for climate-related claims. At the same time, the positive and statistically significant correlation between estimates for ideological bias expression across domains indicates some coherence for ideologically biased responding with regard to the two assessed political topics.

### Study 2 – Empirical testing of theoretical predictions

In Study 2 we empirically tested three theoretical assumptions concerning ideologically biased responding. Specifically, we expected discrimination sensitivity to be relatively independent of ideological bias expression while still showing positive associations with knowledge indicators. We also expected ideological bias expression to be consistent across political issues and to be independently predicted by both political values and party identification.

## Methods

### Participants

Participants were recruited via a German online panel provider (Bilendi GmbH) using quota sampling to approximate population distributions in education, voting behavior, age, and gender. Panelists received monetary compensation for their participation. Data collection took place in December 2024. Of 2,321 panelists who accessed the survey, exclusions were due to non-start (30), dropout (169), failed attention checks (116), ineligibility (3), or fulfilled quotas (618). Detailed quota distributions and population benchmarks are reported in S1A and B Table in the Supporting Information. After additionally removing speeders (top 2% fastest completion times), the final analytic sample comprised *N*_*Study2*_ = 1,359 participants (*M*_*age*_ = 47.68, *SD*_*age*_ = 14.9; 49.5% female). Median completion time was 20.45 minutes.

### Procedure

Participants first provided informed consent, including the voluntary nature of participation, their right to withdraw at any time without penalty, and the anonymous handling of their data. Following consent, they completed demographic questions (age, gender, and education) and the revised 20-item randomized IBE task. Participants then completed measures of political knowledge, political orientation, political values, and party identification. Upon completion of the study, participants were debriefed about the purpose of the research.

### Measures

**Ideological Bias.** The IBE scale for Study 2 was comprised of 20 statements on two political domains: migration/domestic security and climate change/renewable energy. The iterated scale still balances its claims for correctness and ideological congruence of the correct response (Task-Left vs. Task-Right). The instruction and response format were consistent with Study 1, requiring participants to classify each statement as true or false.

**Political Values.** We used an adapted version of the Political Orientation Scale (POS; [64]) measuring five value dimensions with two items each, namely sustainability (e.g., “Climate change worries me”; *α* = .79), economic and personal security (e.g., “Security is a prerequisite for a functioning society”; *α* = .72), tradition (e.g., “Politics should work to preserve traditions”; *α* = .84), anti-immigration (e.g., “It worries me that more and more foreigners come into the country”; *α* = .86) and solidarity (e.g., “There would be fewer problems if everyone were treated equally”; *α* = .63).

Participants responded to each item by indicating approval on a 6-point Likert scale, corresponding to 1—complete disagreement—to 6—complete agreement.

**Party Identification.** A single-item question assessed a culturally specific, long-term partisan affiliation: “In Germany, many people tend towards one party over a prolonged period of time, even while sometimes voting for another party. Do you, in the most general sense, tend to one party? If so, which one?” This item captures the tendency of individuals to consistently favor a particular party over time, regardless of occasional deviations in voting behavior. Respondents could select from all major German political parties.

For the regression analyses, party identification was recoded into a continuous left–right scale reflecting ideological positioning. The linear positioning of the German parties along this scale follows established ideological placements ([65–67]; see S9 Table in the Supporting Information for more details on the coding procedure). Respondents identifying with DIE LINKE were coded as −2 (far left), those identifying with SPD or Bündnis 90/Die Grünen as −1 (moderate left), respondents reporting no or other party identification (e.g., BSW, other/none) as 0, those identifying with CDU/CSU or FDP as +1 (moderate right), and those identifying with AfD as +2 (far right).

## Results

**Fig 1:**
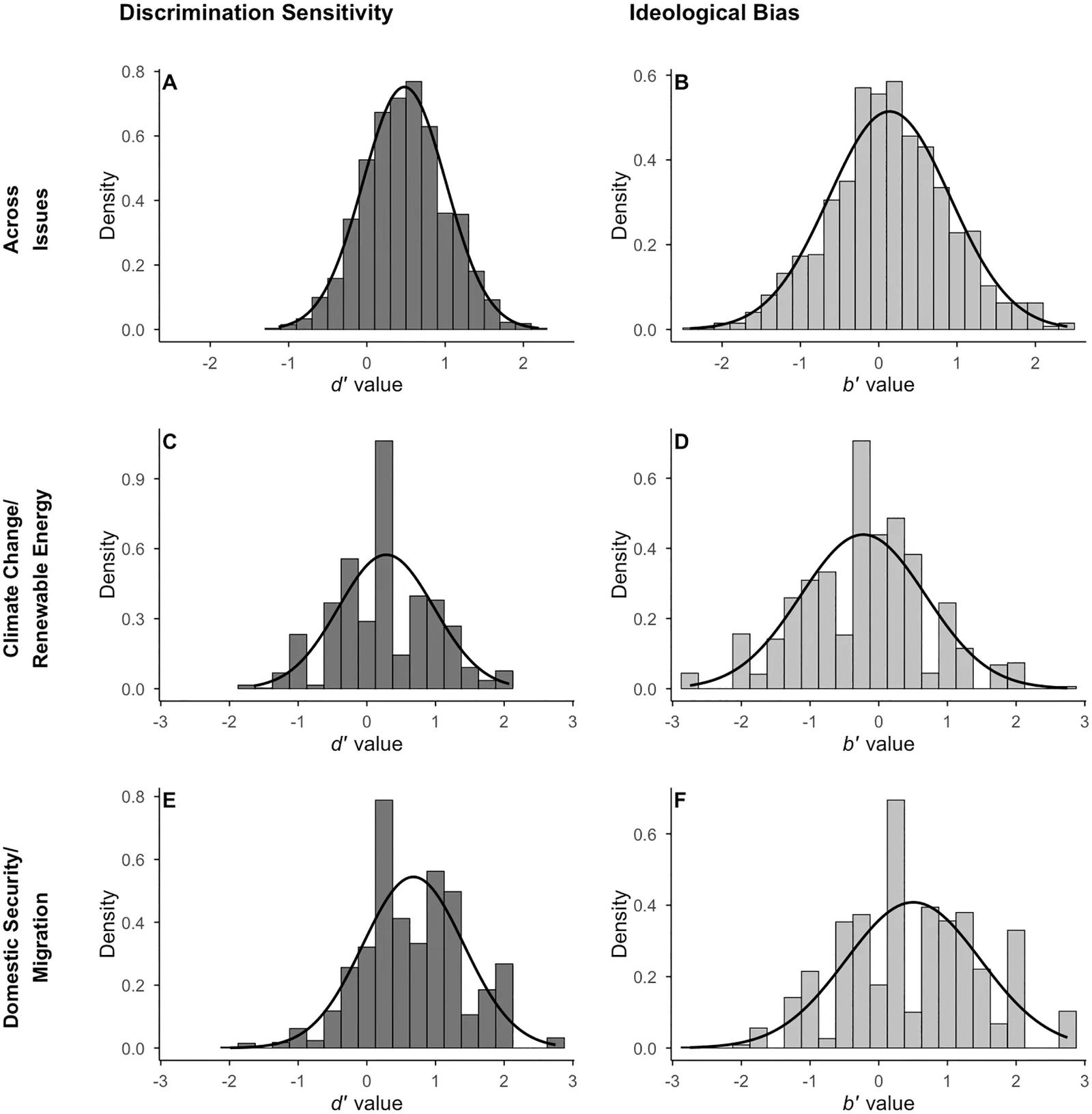
Distributions of Discrimination Sensitivity and Ideological Bias Expression.

We first calculated descriptive statistics for the estimates of discrimination sensitivity and ideological bias expression within domains (climate change/renewable energy and domestic security/migration) and for the overall scale. The density distributions for the *d’* (Panels A, C, E) and *b’* (Panels B, D, F) for the two issue domains climate change/renewable energies, domestic security/migration, and for the overall scale across both domains are reported in Fig 1.

Sample means indicated a lower discrimination sensitivity (*d’* = 0.28, *SD* = 0.69) for the climate change/renewable energy topic compared to the security/migration topic (*d’* = 0.68, *SD* = 0.73). Ideological bias was generally left-leaning (*b’* = −0.23, *SD* = 0.91) in the climate change/renewable energy topic and generally right-leaning (*b’* = 0.50, *SD* = 0.98) for the domestic security/migration topic. Across domains, the overall discrimination sensitivity was moderate (*d’* = 0.48, *SD* = 0.53), likewise, the estimate for across-domain ideological bias expression showed a slight right-leaning tendency on average (*b’* = 0.30, *SD* = 0.62).

Panels show distributions of estimates for discrimination sensitivity (*d*’) and ideological bias expression (*b’*) across issue domains (A–B), for climate change/renewable energy (C–D), and for domestic security/migration (E–F). Each histogram displays the empirical distribution of scores along with a fitted normal density curve. Higher *d*’ values indicate greater discrimination sensitivity, whereas *b’* values reflect bias direction and magnitude. Figure created by the authors.

That is, while participants were slightly better at distinguishing true from false claims in the security-domain compared to the climate change-domain, they nevertheless showed a slight right-leaning bias in the security domain compared to a slight left-leaning bias in the climate domain with regard to their absolute average values. Such a pattern can arise when overall accuracy is higher for security than for climate claims, but the comparatively few errors in the security domain occur disproportionately on items with left-leaning correct response options. For the climate topic, however, this difference was less pronounced. Across all questions and both topics, the sample thus showed a slight right-leaning bias and fair discrimination sensitivity (see Table 6).

**Table 6:** Study 2: Intercorrelations Among Estimates for Ideological Bias Expression and Discrimination Sensitivity.

Variables	*M (SD)*	*b’*_*CC*_	*b’*_*DS*_	*d’*_*M*_	*d’*_*CC*_	*d’*_*DS*_
*b’*_*M*_	.14 (.78)	.81	.84	−.17	−.17	−.08
*b’*_*CC*_	−.23 (.91)		.35	−.24	−.23	−.13
*b’*_*DS*_	.50 (.98)			−.04	−.06	−.01
*d’*_*M*_	.48 (.53)				.73	.76
*d’*_*CC*_	.28 (.69)					.10
*d’*_*DS*_	.68 (.73)					

*Note. N*_*Study2*_ *= 1359; b’ = ideological bias expression; d’ = discrimination sensitivity; CC = issue domain climate change/renewable energy; DS = issue domain domestic security/migration.*

### Relations between Ideological Bias Expression and Knowledge Indicators (RQ1)

Pearson correlations were used to assess the relationships between estimates of ideological bias expression and three indicators of knowledge, namely discrimination sensitivity (*d’*), political knowledge, and education level. To distinguish between the direction and the magnitude of ideological bias expression, we analyzed both the signed *b′* scores, which retain the direction of the bias, and their absolute values (|*b′*|), which capture its magnitude irrespective of direction.

For directional bias, discrimination sensitivity was weakly negatively related to bias in the climate change domain (*r*_*CC*_ = −.24, *p* < .001), but unrelated in the security domain (*r*_*DS*_ = −.04, *p* = .105). Consistent with our findings in Study 1, political knowledge was weakly negatively correlated with directional bias within the climate change domain (*r*_*CC*_ = −.16, *p* < .001), while a negligible association was found within the security domain (*r*_*DS*_ = −.06, *p* = .019). Higher education level was also weakly negatively correlated with directional ideological bias expression in both domains (*r*_*CC*_ = −.16, *p* < .001; *r*_*DS*_ = −.23, *p* < .001).

When considering the magnitude of ideological bias expression (|*b’*|), findings suggest inconsistent relations between the strength of bias and knowledge-indicators. The domain specific estimates for discrimination sensitivity showed weak associations with bias magnitude in opposite directions (*r*_*CC*_ = .07, *p* = .011; *r*_*DS*_ = −.07, *p* = .010) and a weak negative relationship overall (*r*_*M*_ = −.09, *p* = .001). Political knowledge, in contrast, was weakly positively correlated with overall bias magnitude (*r*_*M*_ = .15, *p* < .001) and within the climate change domain (*r*_*CC*_ = .14, *p* < .001), while the association in the security domain was negligible (*r*_*DS*_ = .05, *p* = .046). Education level was essentially unrelated to overall bias magnitude (*r*_*M*_ = .01, *p* = .722).

Taken together, these findings indicate weak and inconsistent relationships between knowledge-related variables and the strength of bias expression, although a slight link between higher knowledge and left-leaning bias expression emerged specifically within the climate change domain.

### Coherence of Ideological Bias Across Assessed Domains (RQ2)

We examined the intercorrelation between domain-specific bias expression estimates to assess the coherence of ideological bias expression across politically polarized issues. *b’*_*CC*_ in the climate domain and *b’*_*DS*_ in the security domain were positively correlated (*r* = .35, *p* < .001), indicating a moderate degree consistency in directional bias expression across both domains. When examining the magnitude of bias expression the correlation between |*b’|* in the two domains was small (*r* = .10, *p* < .001), suggesting that while individuals tend to show consistent directional leanings across domains, the strength of their biased responding varies more substantially by issue.

The results indicate moderate consistency across the assessed domains for the direction and less for the magnitude of the ideological bias expression index. In other words, individuals who displayed a left- or right-leaning bias expression in the climate change domain were likely to show the same directional alignment in the security domain, even though the intensity varied.

### Party Identification and Political Values as Independent Predictors of Ideological Bias Expression (RQ3)

Hierarchical linear regression models examined whether party identification and ideological beliefs independently predicted the ideological bias expression index. Model 1 included age and sex as control variables (see Table 7 for model summaries). This baseline model did not explain a significant proportion of variance (*R^2^* = .004, *p* = .118). Age showed a small positive association with bias, whereas sex was not a significant predictor.

**Table 7:** Summary of hierarchical regression models for predicting ideological bias expression (*b’*).

Predictor	Model 1 *β* 95% CI [LL, UL]	Model 2a *β* 95% CI [LL, UL]	Model 3a *β* 95% CI [LL, UL]
Age	.065* [0.000, 0.007]	.065* [0.001, 0.006]	.033 [-0.001, 0.004]
Sex	.011 [-0.080, 0.113]	.030 [-0.035, 0.129]	.062** [0.026, 0.169]
Party ID (L–R)	—	.530*** [0.275, 0.335]	.183* [0.072, 0.139]
Sustainability	—	—	−.286*** [−0.227, −0.157]
Security	—	—	−.035 [−0.111, 0.022]
Tradition	—	—	.174*** [0.091, 0.180]
Anti-Immigration	—	—	.299*** [0.153, 0.234]
Solidarity	—	—	−.006 [−0.045, 0.035]
Model Fit	*R²* = .004	*R²* = .285	*R²* = .469
Fit Change	—	*ΔR²* = .281 F(1, 1001) = 392.4***	*ΔR²* = .184 F(5, 996) = 69.0***

Note. N = 1005 (listwise after excluding “Other/None” and “BSW”); β = standardized regression weight; CI = 95% confidence interval for B. Significance codes. p < .10, * p < .05, ** p < .01, *** p < .001.

In Model 2a, party identification was entered as a continuous left–right variable ranging from −2 (far left) to +2 (far right). Adding party identification significantly increased explained variance (*ΔR^2^* = .281, *F*(1, 1001) = 392.38, *p* < .001), resulting in *R^2^* = .285. Party identification showed a strong positive association (*β* = .530, *p* < .001), indicating that identification with the political right was associated with more right-leaning bias expression scores and vice versa. The effect held under an alternative categorical specification of party identification, using respondents without a party preference or aligned with other parties as the reference group.

In Model 3a, political value orientations (sustainability, security, tradition, anti-immigration, and solidarity) were added to examine whether ideological beliefs explain variance beyond party identification. This step significantly improved model fit (*ΔR^2^* = .184, *F*(5, 996) = 68.95, *p* < .001), yielding a total *R^2^* = .469. Sustainability (*β* = −.286, *p* < .001) was associated with more left-leaning bias scores, whereas tradition (*β* = .174, *p* < .001) and anti-immigration (*β* = .299, *p* < .001) were associated with more right-leaning bias scores. Security and solidarity were not significant predictors. Importantly, party identification remained a significant predictor in the full model (*β* = .183, *p* < .001), but its effect size was substantially reduced compared to Model 2a, indicating considerable shared variance between ideological identity and value orientations.

To better compare the relative contributions of ideological beliefs and party identification, the hierarchical regression was repeated in reversed order. In Model 2b, political value orientations were entered after the control variables (see S8 Table in the Supporting Information for model summaries of the reversed hierarchical regressions). This step accounted for a substantial increase in explained variance (*ΔR^2^* = .444, *F*(5, 997) = 115.90, *p* < .001), resulting in *R^2^* = .449. Sustainability (*β* = −.336, *p* < .001), tradition (*β* = .200, *p* < .001), and anti-immigration (*β* = .371, *p* < .001) emerged as significant predictors, whereas security and solidarity were not significant. In Model 3b, party identification was added to the model. Although this addition led to a statistically significant improvement in model fit (*ΔR^2^* = .020, *F*(1, 996) = 37.45, *p* < .001), the incremental variance explained was comparatively small, yielding a final *R^2^* = .469. Party identification remained a significant predictor (*β* = .183, *p* < .001), but its contribution was modest relative to the variance explained by value orientations.

## Discussion

In Study 2, findings from Study 1 were extended by validating the IBE scale and testing key theoretical predictions about the psychological underpinnings of ideological bias. First, ideological bias expression showed weak and inconsistent associations with a measure of political knowledge for a German political context (HIP) and formal education. Also participants’ ability to accurately differentiate between true and false claims was not meaningfully associated with less bias expression. The internal consistency of the HIP was only moderate, implying that the observed associations may represent conservative estimates. However, this limitation is unlikely to account for the overall pattern of findings.

Second, bias expression estimates were moderately positively intercorrelated between the assessed issue domains, providing initial evidence that ideological bias is not domain-specific. Specifically, individuals with stronger left- or right-leaning bias expression in the climate change domain were also more likely to exhibit a similarly aligned bias in the security domain.

Finally, Study 2 showed that both political values and party identification independently predicted ideological bias expression estimates, each explaining unique variance in the regression models. However, political values accounted for substantially more variance, and the effect of party identification was notably attenuated once value orientations were included. Although the comparatively lower reliability of the solidarity scale may have attenuated its association with the estimate for bias expression, the remaining value dimensions (sustainability, tradition, and anti-immigration) consistently emerged as strong predictors, suggesting that the overall pattern cannot readily be attributed to differences in measurement precision alone. Taken together, these findings suggest that ideological bias is primarily shaped by underlying political values, with party identification exerting a smaller yet still independent influence, indicating partial overlap but not redundancy between these constructs in the German context.

### General Discussion

In the present research, we conceptualized ideological bias expression as the individual tendency to selectively accept or reject scientific evidence depending on its alignment with ideological beliefs or political affiliations. We developed and validated the Ideological Bias Expression (IBE) scale to capture interindividual differences in biased evaluation of evidence across two highly polarized domains: climate change/renewable energy and domestic security/migration. Using a signal detection framework, our approach quantifies ideological bias expression as within-person asymmetries in accuracy as a function of ideological congruence of correct response options.

We used this measure to test three theoretical assumptions about the nature of ideologically biased evaluation of evidence (RQ1–RQ3). Results showed (a) no consistent association between either the direction or magnitude of ideological bias expression and knowledge-related indicators, (b) moderate coherence in the direction of bias expression across two assessed issue domains and (c) independent contributions of political identity and political value orientations in predicting estimates of bias expression.

### Psychometric properties of the ideological bias expression scale

Psychometric analyses support the scale’s utility as an effective tool for capturing ideological bias in the evaluation of evidence. The IBE scale’s 20 items met psychometric standards regarding difficulty and item-scale discrimination. Item-scale correlations were consistent with theoretical expectations, indicating that performance on ideologically aligned items systematically contributes to the expected direction of bias. We gathered additional evidence for the validity of bias estimates based on empirical relations with political identity and political values. In our studies, approximately 45% of the variance in ideological bias expression can be explained by political identity and political values.

The split-half procedure indicated acceptable internal consistency for estimates of ideological bias expression. Relative to reliability estimates reported for several cognitive task paradigms, this level of reliability appears comparable or favorable (e.g., [68]). In contrast, discrimination sensitivity exhibited low split-half reliability, limiting conclusions about individual differences in discrimination accuracy and requiring cautious interpretation of correlations involving *d′*.

At the same time, split-half reliability should be interpreted cautiously for SDT-derived parameters like *d′* as it is not a sum score of interchangeable items but a nonlinear estimate derived from hit and false-alarm rates. Splitting the task into halves necessarily reduces the number of signal and noise trials available for estimating these rates, thereby increasing estimation error and attenuating split-half reliability.

Moreover, high item difficulty lead to discrimination performance nearing chance level, resulting in reduced variability in discrimination sensitivity, limiting split-half reliability of *d’*. Importantly, item difficulty was intentionally high as this provided greater sensitivity to ideological distortions, thereby being favorable for the primary function of estimating bias expression. Nevertheless, future research should aim to improve the precision of *d′* by increasing the number of trials. In addition, using repeated measurements may provide a more informative assessment of the stability of discrimination sensitivity than internal consistency estimates alone.

A critical step during scale construction was to rule out that unbalanced item properties affect our measure of ideological bias expression. Robustness analyses consistently suggested that the estimates of ideological bias expression were not driven by differences in item properties. Instead, the probability of correctly evaluating a claim depended systematically on the congruence between its ideological alignment and participants’ self-rated political orientation. The effect remained stable after accounting for differences in item difficulty, item-specific ideology effects and overall response accuracy. This supports the interpretation of ideological bias as a systematic asymmetry in the evaluation of evidence rather than an artifact of the measurement instrument.

Unlike classical SDT applications, our measure requires evaluating the veracity of politically charged claims. The complexity and motivational relevance of these stimuli challenges the assumption of stable internal decision criteria across items. Depending on whether information is ideologically congruent or incongruent, participants may shift between processing styles, relying more on systematic or affective styles of information processing, depending on the specific item. Evaluations within our decision-tasks may therefore include not only systematic epistemic evaluations but potentially low-effort intuitive or even expressive or strategic responding (e.g., [12]).

We analyzed low-effort responding as an indicator of intuitive responding by eliminating the fastest response times and found that key estimates remained virtually unchanged, suggesting that results are not driven by rapid intuitive responding. While our indirect measurement approach makes strategic responding cognitively demanding, this kind of response behavior remains plausible.

Importantly, our measure should be interpreted as a behavioral index of ideological bias expression. The present design does not allow us to determine which cognitive or motivational mechanisms underlie the observed asymmetries. Future validation studies could address this limitation by incorporating response times, confidence ratings, or accuracy incentives to examine the role of processing depth and deliberation. In addition, source manipulations and process-tracing methods could help distinguish whether biased evaluations are more consistent with motivated reasoning, identity-protective processes, or cognitive mechanisms such as heuristic information processing.

### Dissociations between ideological bias expression and knowledge indicators

We found preliminary evidence of a weak empirical relationship between political knowledge and ideological bias. Across both issue domains, relationships between ideological bias expression indices and different knowledge indicators (discrimination sensitivity, political knowledge, formal education) were weak and inconsistent. In the domain of national security/migration, the estimate for directional ideological bias expression was essentially unrelated to all three indicators of knowledge. In the climate change domain, small negative associations emerged, suggesting that higher levels of knowledge were weakly related to reduced directional bias. However, these effects were weak and not consistently observed across indicators. A similarly inconsistent pattern was observed for the magnitude of ideological bias expression. Associations between knowledge indicators and bias strength were consistently small and varied in direction across domains. Taken together, these findings provide little evidence for a systematic relationship between political knowledge and either the direction or the strength of ideological bias expression. It should be noted, that moderate to low internal consistency of both the political knowledge measure and discrimination sensitivity is likely to attenuate the observed associations. Consequently, any true associations may be somewhat stronger than those observed here, although the overall pattern remains inconsistent and provides little evidence for a robust relationship.

This disconnect between indices for political knowledge and ideological bias expression is not in line with the theoretical claim that ideological bias reflects a lack of knowledge or reasoning skills [28], which would suggest a negative relation between knowledge and bias. Instead, a plausible interpretation builds on the framework of motivated reasoning (e.g., [26]). In this logic, ideological bias reflects the selective deployment of reasoning to defend preferred conclusions, operating not despite thematic competence but often because of it. In other words, individuals can exhibit high discrimination accuracy or political knowledge while still displaying ideologically skewed judgments, highlighting the role of selective reasoning in service of identity or value alignment [44,69].

### Coherence of Ideological bias expression across assessed domains

Our findings support the assumption that ideologically biased evaluation of evidence is not purely domain-specific, but reflects a coherent tendency across domains. The moderate correlations between bias estimates across two issues are consistent with accounts proposing a domain-general propensity toward ideologically motivated cognition [42,43]. In this sense, our results speak against a strictly “innocent” view of ideology, as articulated by Kinder and Kalmoe [37], and the idea that most citizens lack a deep, overarching ideological structure. Our findings rather suggest that individuals who evaluate evidence in a biased manner in one domain are more likely to show the same directional alignment for bias in another distinct domain.

However, the observed coherence is far from perfect. Rather than supporting a fully coherent ideological belief system, the pattern and magnitude of the associations point toward the influence of both domain-general dispositions (e.g., motivated reasoning, identity-protective cognition) and by domain-specific factors such as issue salience or personal relevance. This interpretation aligns with the broader literature suggesting that political cognition in laypersons is structured (e.g., [40]), but only loosely constrained by overarching ideological frameworks.

### Political identity and political values predict ideological bias expression

Ideological bias was predicted by party identification and political values, with each explaining unique variance in the regression models. This is notable given the conceptual overlap between the two constructs, especially in political environments where party cues often serve as proxies for ideological positions. The fact that both predictors remained significant when entered simultaneously suggests that they capture partially distinct mechanisms underlying biased evaluation of evidence.

The findings indicate that ideological bias might be more closely tied to individuals’ broader value commitments. Political values likely provide a framework through which evidence is evaluated, shaping what is perceived as credible, relevant, or normatively desirable. In contrast, party identification, while still a significant predictor, appears to exert a comparatively weaker influence in this context. One possible interpretation is that political values reflect more deeply internalized orientations that guide reasoning even in the absence of explicit partisan signals. This is especially informative in light of ongoing debates, particularly in the U.S. literature, about whether ideological bias is primarily driven by social identity processes linked to party affiliation [44]. From that perspective, biased evaluation of evidence is often understood as a form of identity protection, where individuals align their judgments with the perceived positions of their in-group. Our findings only partially support this account. While party identification does play a role, the stronger and more consistent effects of political values suggest that ideological bias cannot be reduced to partisan identity alone.

Importantly, the present results emerge from a multi-party context, where party affiliation and ideological value orientations are less tightly coupled than in two-party systems. This institutional setting provides a more stringent test of the relative contributions of identity-based and value-based explanations. The observed independence of value effects therefore strengthens the argument that ideological bias is, at least in part, rooted in underlying value commitments rather than merely reflecting partisan alignment. Individuals who share similar values may exhibit similar patterns of biased evaluation even when they do not identify with the same political party. More broadly, our findings align with theoretical models that conceptualize political reasoning as jointly shaped by identity-based and value-based processes (e.g., [43]). Rather than viewing these explanations as competing, our results suggest party identification may amplify or channel biases in contexts where group identity is salient, whereas political values provide a more general basis for interpreting information across contexts.

### Limitations

Several limitations should be considered in the present research. First, the item set was derived from contemporary empirical sources, introducing constraints on temporal stability. The measure relies on a set of heterogeneous items, based on potentially time-sensitive empirical evidence (e.g., current statistics or scientific reports). Even with a maintenance procedure in place (see S3 Table in the Supporting Information), their validity may change over time, potentially limiting the longitudinal comparability of the measure.

Second, our measure comprises factual claims from two issue domains, specific to the German political context, and generalization beyond these domains and contexts requires further validation. Demonstrating consistent individual differences across two substantively distinct and politically salient domains provides initial evidence that ideologically biased evaluation is not specific to single issue areas. Nonetheless, the two domains examined in the present study represent only a narrow subset of the issue space typically used to operationalize political ideology [70]. As such, the observed patterns in relation to bias observed here may not generalize to domains that are less clearly aligned along a single ideological dimension, or that vary in moralization, complexity, or partisan cueing. Future research should therefore examine a broader and more heterogeneous set of issues, to more precisely estimate the extent and limits of cross-domain coherence.

Third, the measure is embedded in the context of contemporary German political discourse. The ideological alignment of items was defined based on issue-specific positions prevalent in Germany, and the interpretation of claims may depend on culturally and politically shared frames of reference. This context-specificity limits the direct transferability of the findings to other countries or political systems, where both the content of factual claims and their ideological implications may differ. Accordingly, when adapting the task to other countries or issue domains, the ideological alignment of items should ideally be established through independent pretesting.

Taken together, these limitations highlight the trade-offs inherent in the issue-specific construction of items. Future research could address some constraints by expanding the item pool and incorporating additional issue domains to assess the stability and breadth of ideological bias across different political and cultural contexts.

### Practical implications

The present findings have implications for the design of interventions aimed at reducing ideological bias. Ideological bias seems to be persistent also among individuals with relatively high discrimination accuracy and political knowledge. Consequently, approaches that focus solely on improving discriminative ability, such as accuracy nudges that are intended to enhance analytical thinking and knowledge under cognitive accounts (e.g., [33]), may have limited effects. This challenges interventions based purely on cognitive accounts, which assume that better knowledge and reasoning reduces bias, and suggests that increasing competence alone does not necessarily translate into more balanced evaluations.

From a motivated reasoning perspective, ideological bias being predicted independently by both political identity and ideological values, implies that identity-focused interventions, such as those based on motivational accounts (e.g., [34]), may need to address both social identity and value-based ideological convictions. Debiasing strategies, especially in political contexts where party identification and political values are not perfectly aligned, may benefit from addressing multiple motivational sources, such as fostering cross-cutting social identities, reframing issues in value-congruent ways, or promoting reflective dialogue that acknowledges competing moral foundations.

Finally, our measure allows for a better empirical evaluation of the effectiveness of interventions aimed at reducing ideological bias. Whereas previous studies analyzed group differences (e.g., [49,50]), our measure provides the opportunity to investigate interindividual differences in the effectiveness of interventions.

## Conclusion

This research addresses a central measurement gap in the study of ideological bias: the reliance on ad hoc instruments with unclear reliability and validity, as well as the frequent conflation of ideological bias with ideological stance. These limitations hinder the systematic study of ideological bias and the evaluation of interventions aimed at reducing it. To overcome this, we introduce the Ideological Bias Expression (IBE) scale, a measure of individual differences in ideological bias expression during the evaluation of evidence, grounded in signal detection theory.

Across two German samples, our measure suggests little evidence for a systematic relationship between ideological bias expression and knowledge-related indices, including education and political knowledge. These findings are consistent with the view that ideological bias is not simply a function of differences in knowledge or reasoning ability, in line with dual-process accounts of motivated reasoning. In addition, a moderate coherence of ideological bias expression across two distinct issue domains is initial evidence that biased evaluation of evidence is not limited to specific issue domains. Finally, our research suggests that ideological bias cannot be reduced to partisan bias, but reflects a combination of social identity dynamics and value-based cognition, with the latter playing a central role in shaping how individuals evaluate evidence in a multi-party environment.

Beyond its theoretical implications, the IBE scale provides a tool for disentangling discrimination ability and biased responding during evidence evaluation and offers a foundation for future research on the determinants of ideological bias and potential interventions to promote more evidence-based public discourse.

## Supporting information

S1 Table Distribution of final Sample across Quota Categories and Population Benchmarks. (PDF)

S2 Table IBE Items with References. (PDF)

S3 Table Maintenance Framework for IBE Items. (PDF)

S4 Table Logistic Regression Predicting Candidate Item Responses from Political Knowledge and Political Orientation (Study 1). (PDF)

S5 Table Split-Half Reliability for Discrimination Sensitivity and Ideological Bias Index. (PDF)

S6 Table Task-Specific Difficulty and Discrimination Distributions by Domain. (PDF)

S7 Table Summary of Multilevel Logistic Regression Predicting Probability of Correct Responses. (PDF)

S8 Table Summary of Reversed Hierarchical Regression Models for predicting Ideological Bias Expression (b’). (PDF)

S9 Table Mapping of Political Party Ideological Positions to Coding Categories Based on MARPOR RILE and CHES Left–Right Expert Scores. (PDF)

S1 Fig Interaction between Ideology and Item Congruence on the Probability of Correct Responses. (PDF)
