# Supplementary material for: Ideologically biased evaluation of evidence - A signal-detection approach to measure individual differences
Source: PLoS One. 2026 Sep 11;21(9):e0357011. doi: 10.1371/journal.pone.0357011 (PMC13568519; doi:10.1371/journal.pone.0357011)
Supplement: Supplementary file 1 [file pone.0357011.s001.pdf]

Table S1A. Distribution of final Sample for Study 1 across Quota Categories and Population Benchmarks.

Quota Name	Bracket	Population Benchmark (%)	Target Sample (*n*)	Completed Responses (*n*)
Education status 1	Lower secondary or less	32.94	171	99
Education status 2	Intermediate secondary	30.56	159	159
Education status 3	Upper secondary / tertiary	36.50	190	198
Voting behavior 1	CDU/CSU	21.64	113	104
Voting behavior 2	SPD	20	104	103
Voting behavior 3	AfD	7.67	40	44
Voting behavior 4	FDP	6.61	34	34
Voting behavior 5	DIE LINKE	3.77	20	17
Voting behavior 6	Bündnis 90/ Die Grünen	10.57	55	55
Voting behavior 7	Nichtwähler, Nicht-Wahlberechtigt, Sonstige	29.74	155	99
Age group 1	18-29 (male)	8.29	43	18
	18-29 (female)	7.63	40	31
Age group 2	30-39 (male)	8.09	42	37
	30-39 (female)	7.74	40	40
Age group 3	40-49 (male)	7.26	38	38
	40-49 (female)	7.28	38	39
Age group 4	50-59 (male)	9.13	47	47
	50-59 (female)	9.14	48	48
Age group 5	+59 (male)	16.07	84	84
	+59 (female)	19.36	101	73
Total *n*			520	456

Note. German online panel sample (provider: Cint Deutschland GmbH; fielded July 2024). Percentages for Population benchmark reflect distributions used for quota targets. Education brackets were defined as follows: Education status 1 (Lower secondary or less) = currently in school, no school-leaving certificate, or Haupt-/Volksschulabschluss; Education status 2 (Intermediate secondary) = Realschulabschluss or equivalent; Education status 3 (Upper secondary/tertiary) = Fachhochschulreife, Hochschulreife (Abitur), or tertiary degree (Bachelor, Master/Diplom, Doctorate, Habilitation). Population benchmarks were derived from Destatis Microcensus, Eurostat population statistics, and official results of the 2021 German federal election.

Study 1

Participants were recruited via a German panel provider (Cint Deutschland GmbH). For the data collection process of Study 1 quotas were implemented to approximate population distributions, as reported in Table S1. Education quotas distinguished between three levels of educational status, aligning with German school qualifications. Political affiliation quotas included supporters of major German parties as well as non-voters, individuals not eligible to vote, and supporters of other parties. Age and gender were cross-quoted across five age groups, with separate targets for male and female participants.

In July 2024, a total of 1,409 panel members accessed the survey link. Of these, 17 did not start the questionnaire, 16 discontinued participation before completion, and 25 participants failed an attention check. One respondent was younger than 18 years and was excluded. During fielding, 894 panelists were screened out because the quota category they belonged to had already been filled.

The distribution of completed responses (n = 456) across quota categories is reported in Table S1A, together with population benchmarks from German population statistics (Destatis Microcensus (2023) and federal 2021 election statistics. Among completed responses 10 speeders (top 2% fastest completion times) were removed. The final analytic sample therefore consisted of N_{Study1} = 446 participants (M_{age} = 50.47, SD_{age} = 14.5, 50.45% female). Participants completed the survey in a median time of 12.12 minutes.

Table S1B. Distribution of final Sample for Study 2 across Quota Categories and Population Benchmarks.

Quota Name	Bracket	Population Benchmark (%)	Target Sample (*n*)	Completed Responses (*n*)
Education status 1	Lower secondary or less	32.94	494	377
Education status 2	Intermediate secondary	30.56	458	460
Education status 3	Upper secondary / tertiary	36.50	548	548
Voting behavior 1	CDU/CSU	21.64	501	470
Voting behavior 2	SPD	20	225	225
Voting behavior 3	AfD	7.67	278	278
Voting behavior 4	FDP	6.61	60	60
Voting behavior 5	DIE LINKE	3.77	50	46
Voting behavior 6	Bündnis 90/ Die Grünen	10.57	174	143
Voting behavior 7	Nichtwähler, Nicht-Wahlberechtigt, Sonstige	29.74	213	163
Age group 1	18-29 (male)	8.29	124	126
	18-29 (female)	7.63	114	115
Age group 2	30-39 (male)	8.09	121	124
	30-39 (female)	7.74	116	117
Age group 3	40-49 (male)	7.26	109	109
	40-49 (female)	7.28	109	109
Age group 4	50-59 (male)	9.13	137	137
	50-59 (female)	9.14	137	138
Age group 5	+59 (male)	16.07	241	208
	+59 (female)	19.36	290	202
Total *n*			1500	1385

Note. German online panel sample (provider: Bilendi GmbH; fielded December 2024). Percentages for Population benchmark reflect distributions used for quota targets. Education brackets were defined as follows: Education status 1 (Lower secondary or less) = currently in school, no school-leaving certificate, or Haupt-/Volksschulabschluss; Education status 2 (Intermediate secondary) = Realschulabschluss or equivalent; Education status 3 (Upper secondary/tertiary) = Fachhochschulreife, Hochschulreife (Abitur), or tertiary degree (Bachelor, Master/Diplom, Doctorate, Habilitation). Population benchmarks were derived from Destatis Microcensus, Eurostat population statistics, and official results of the 2021 German federal election.

Study 2

Participants were recruited by a German online panel provider (Bilendi GmbH). Quotas matched those used in Study 1 to approximate population distributions, with proportionally larger allocations to accommodate a higher target sample size: Educational attainment (three levels), voting behavior (major German parties, other parties and non-voters), age group, and gender (cross-quoted across five age groups).

In December 2024, 2,321 panel members accessed the survey link. Of these, 30 did not start the questionnaire, 169 discontinued participation before completion, and 116 participants failed an attention check. Three respondents were younger than 18 years and were therefore excluded. During fielding, 618 panelists were screened out because the quota category they belonged to had already been filled. The distribution of completed responses (n = 1384) across quota categories is reported in Table S1B**,** together with population benchmarks from German population statistics (Destatis Microcensus (2023) and federal 2021 election statistics. Among completed responses 26 speeders (top 2% fastest completion times) were removed. The final analytic sample therefore consisted of N = 1,359 participants. The median survey completion time was 20.45 minutes (M_{age} = 47.68, SD_{age} = 14.9), and 49.5% of participants identified as female.
