# Supplementary material for: Ideologically biased evaluation of evidence - A signal-detection approach to measure individual differences
Source: PLoS One. 2026 Sep 11;21(9):e0357011. doi: 10.1371/journal.pone.0357011 (PMC13568519; doi:10.1371/journal.pone.0357011)
Supplement: Supplementary file 2 [file pone.0357011.s002.pdf]

Table S2. IBE Items with References.

No.	Item Text: German Original	Item Text: English Translation	ID	Truth Value of Claim	Empirical Evidence	Empirical Source	Ideological Congruence of Correct Response	Ideological Framing Source
1	Obwohl in Deutschland 1,1% der Weltbevölkerung lebt, beträgt der deutsche Anteil am weltweiten CO2 Ausstoß weniger als 1%.	Although 1.1% of the global population lives in Germany, the German share of global carbon emissions is below 1%.	CC	False	Germany's share of global CO_2 emissions relative to its share of global population (cross-sectional comparison).	Friedlingstein, P., et al. (2023). Global carbon budget 2023. Earth System Science Data.	Task-Left	Häusler, A. (2019). Die AfD und der Klimawandel: Zwischen Leugnung und national-ökologischer Wende. Heinrich-Böll-Stiftung.
2*	Ein Auto mit Benzinmotor verursacht im Vergleich mit einem E-Auto über seinen gesamten Lebensweg hinweg wesentlich mehr Treibhausgase.	A car with a gasoline engine produces significantly more greenhouse gases over its entire lifecycle compared to an electric car.	CC	True	Lifecycle greenhouse-gas emissions comparison between electric and gasoline vehicles.	ADAC (2022). Lebenszyklusanalyse-Tool (LCA).	Task-Left	AfD-Fraktion im Deutschen Bundestag (2019). Kleine Anfrage: Gegenwind für das E-Auto. Drucksache 19/12231.
3	Die Sommer-Eisfläche der Arktis war 1980 noch mehr als ein Drittel größer als heute.	The summer ice-covered surface of Antarctica was about one-third larger in 1980, compared to today's.	CC	True	Arctic summer sea ice extent comparison (1980 vs. present).	Alfred-Wegener-Institut & Universität Bremen (2020). Meereisportal trend curves.	Task-Left	Greenpeace Deutschland. (2024). Eisschwund in Arktis und Antarktis.
4	Die führenden 20 Industrie- und Schwellenländer (G20) sind für weniger als ein Drittel des weltweiten Treibhausgasausstoßes verantwortlich.	The 20 leading industrial and threshold countries (G20) are responsible for less than a third of global greenhouse gases.	CC	False	G20 aggregate greenhouse-gas emissions relative to global total.	Intergovernmental Panel on Climate Change (2023). AR6 synthesis report.	Task-Left	Germanwatch. (2021). G20-Staaten verantwortlich für gut 75 Prozent der globalen Emissionen [Press release].
5	Die Nutztierhaltung ist in Deutschland für mehr als 5% der erzeugten Treibhausgase verantwortlich.	Livestock farming in Germany is responsible for 5% of the produced greenhouse gases.	CC	True	Share of livestock-related emissions in Germany's agricultural GHG emissions.	Umweltbundesamt (2022). National inventory report for the German greenhouse gas inventory.	Task-Left	Deutscher Bauernverband (2019). Klimastrategie 2.0. Berlin.
6	Der CO2-Ausstoß pro Kopf in Deutschland ist wesentlich geringer als der Chinas.	Per capita CO2 emissions in Germany are significantly lower than in China.	CC	False	Per-capita fossil CO_2 emissions comparison between Germany and China.	Global Carbon Atlas (2024).	Task-Left	Greenpeace Deutschland. (n.d.). Klimagerechtigkeit.
7*	In Deutschland befinden sich von Jahr zu Jahr weniger Personen in Haft in einer Justizvollzugsanstalt (JVA).	In Germany, the number of people in custody in a correctional facility (JVA) decreases from year to year.	DS	True	Number of incarcerated persons in Germany (long-term time series).	Statistisches Bundesamt (2024). Strafvollzugsstatistik.	Task-Left	Dessecker, A. (2021). Vom Recht und seiner Realität: Strafvollzug in Deutschland. Aus Politik und Zeitgeschichte, 71(42–43), 10–16.
8*	Wohnungseinbrüche haben in Deutschland innerhalb der letzten zehn Jahre abgenommen.	Burglaries in Germany have decreased over the past ten years.	DS	True	Number of recorded burglaries in Germany over the last decade.	Bundeskriminalamt (2024). Polizeiliche Kriminalstatistik 2023.	Task-Left	Mediendienst Integration (2023). Faktencheck Kriminalität.

No.	Item Text		ID	Truth Value of Claim	Empirical Evidence	Empirical Source	Ideological Congruence of Correct Response	Ideological Framing Source
	German Original	English Translation						
9	Die Mehrheit der Tatverdächtigen bei Wohnungseinbrüchen in Deutschland ist deutsch.	The majority of suspects in cases of apartment break-ins in Germany are German.	DS	True	Nationality distribution of burglary suspects (PKS).	Bundeskriminalamt (2024). Polizeiliche Kriminalstatistik 2023.	Task-Left	Mediendienst Integration. (2026). Begehen Ausländer öfter Straftaten als Deutsche?
10*	Heute werden weniger rechtsextreme Personen vom Verfassungsschutz gelistet als noch vor fünf Jahren.	Today, fewer right-wing extremists are listed by the domestic intelligence service than five years ago.	DS	False	Number of right-wing extremists recorded by domestic intelligence (time series).	Bundesamt für Verfassungsschutz (2023). Verfassungsschutzbericht 2022.	Task-Left	Amadeu Antonio Stiftung (2023). Jahresreport Rechtsextremismus.
11	Rechtsextreme Straftaten haben in Deutschland innerhalb der letzten zehn Jahre zugenommen.	Extreme right-wing offences in Germany have increased during the last 10 years.	DS	True	Right-wing extremist offences recorded in Germany (time series).	Bundesamt für Verfassungsschutz (2023). Verfassungsschutzbericht 2022.	Task-Left	Amadeu Antonio Stiftung (2023). Jahresreport Rechtsextremismus.
12	Über 80% der in Deutschland gestellten Asylanträge werden bewilligt.	More than 80% of asylum applications posed in Germany are approved.	DS	False	Overall protection rate of asylum applications in Germany.	Bundesamt für Migration und Flüchtlinge (2024). Bundesamt in Zahlen 2023.	Task-Left	Konrad-Adenauer-Stiftung (2023). Migrationspolitische Debatten.
13	Die Anzahl der durchgeführten Abschiebungen aus Deutschland nimmt seit 2020 jährlich zu.	The number of executed deportations in Germany has been rising annually since 2020.	DS	True	Executed deportations from Germany (2020–2023 time series).	Bundesministerium des Innern und für Heimat (2023). Migrationsbericht.	Task-Left	European Council on Foreign Relations (2023). EU return policy brief.
14*	Die Anzahl der jährlichen Abschiebungen nimmt seit fünf Jahren stetig zu.	The number of annual deportations has been steadily increasing for five years.	DS	True	Five-year time series of deportations executed from Germany.	Bundesministerium des Innern und für Heimat (2023). Migrationsbericht.	Task-Left	European Council on Foreign Relations (2023). Migration policy brief.
15*	In Deutschland haben Personen mit einem Migrationshintergrund weniger häufig die Fachhochschul- oder Hochschulreife als Personen ohne Migrationshintergrund.	In Germany, people with a migration background are less likely to have a university of applied sciences or university entrance qualification than people without a migration background.	DS	False	Educational attainment among individuals with vs. without migration background.	BAMF (2024). Bundesamt in Zahlen 2023.	Task-Left	DeZIM (2024). Integrationsbericht.
16	Seit Beginn der Industrialisierung hat die Erderwärmung 1,8° C nicht überschritten.	Since the beginning of industrialization, global warming has not surpassed 1.8°C.	CC	True	Global mean surface temperature change relative to pre-industrial baseline.	IPCC (2023). AR6 synthesis report.	Task-Right	UNFCCC (2023). COP outcome documents.
17	Der Ausstieg aus der Kernenergie hat in Deutschland zu einem Anstieg der Strompreise geführt.	The withdrawal from nuclear power in Germany has caused a rise in electricity prices.	CC	False	Electricity price trends before and after nuclear phase-out.	Bundesministerium für Wirtschaft und Klimaschutz (2023). Energy data portal.	Task-Right	Friedrich-Naumann-Stiftung (2023). Energy policy papers.

No.	Item Text		ID	Truth Value of Claim	Empirical Evidence	Empirical Source	Ideological Congruence of Correct Response	Ideological Framing Source
	German Original	English Translation						
18*	Der CO2-Ausstoß pro Kopf ist in Deutschland seit 1990 um mehr als ein Drittel gesunken.	Per capita CO2 emissions in Germany have decreased by more than a third since 1990.	CC	True	Per-capita CO_2 emissions change in Germany since 1990.	Umweltbundesamt (2024). Treibhausgas-Emissionen in Deutschland seit 1990.	Task-Right	Expertenrat für Klimafragen (2023). Prüfbericht 2023.
19	Der Ausstoß von Treibhausgasen nimmt in Deutschland immernoch jährlich zu.	Greenhouse gas emissions are still increasing annually in Germany.	CC	False	Total GHG emissions in Germany (time series).	Umweltbundesamt (2024). Treibhausgas-Emissionen Zeitreihe.	Task-Right	Expertenrat für Klimafragen (2023). Annual assessment report.
20	Ein Fahrzeug mit elektrischem Antrieb benötigt bei seiner Herstellung mehr Energie als ein Fahrzeug mit Verbrennungsmotor.	A vehicle with an electric drive requires more energy to produce than a vehicle with an internal combustion engine.	CC	True	Production-phase energy demand comparison between EV and ICE vehicles.	ADAC (2022). Lebenszyklusanalyse-Tool.	Task-Right	Agora Verkehrswende (2022). Klimabilanz von E-Autos.
21	Der Anteil der erneuerbaren Energien am gesamten Energieverbrauch in Deutschland hat sich in den letzten zehn Jahren verdoppelt.	The share of renewable energies in total energy consuptions has doubled in the last 10 years.	CC	True	Share of renewable energy in Germany's gross final energy consumption (time series).	Umweltbundesamt (2024). Zeitreihen erneuerbare Energien.	Task-Right	Agora Energiewende (2024). Energiewende status report.
22	Weniger als jede/r dritte Straftatverdächtige in Deutschland ist Ausländer/in.	Less than every third criminal offender in Germany is a foreigner.	DS	False	Share of non-German suspects among all suspects (PKS).	Bundeskriminalamt (2024). Polizeiliche Kriminalstatistik 2023.	Task-Right	Mediendienst Integration (2026). Begehen Ausländer öfter Straftaten als Deutsche?
23*	Die Anzahl der Drogentoten in Deutschland nimmt seit über zehn Jahren zu.	The number of drug-related deaths in Germany has been increasing for over ten years.	DS	True	Drug-related deaths in Germany (long-term trend).	Bundeskriminalamt (2024). Bundeslagebild Rauschgiftkriminalität.	Task-Right	EMCDDA (2023). European Drug Report.
24	Gewalttaten gegen Polizistinnen und Polizisten nehmen seit über zehn Jahren jährlich zu.	Violent attacks against police officers in Germany have been increasing annually for more than ten years.	DS	True	Violence against police officers in Germany (time series).	Bundeskriminalamt (2023). Gewalt gegen Polizeivollzugsbeamt*innen – Bundeslagebild.	Task-Right	Deutsche Polizeigewerkschaft (2023). Positionspapier Gewalt gegen Polizei.
25*	Von den 2022 erfassten linksextremistischen Straftaten in Deutschland war weniger als jede Zehnte eine Gewalttat.	Of the left-wing extremist crimes recorded in Germany in 2022, less than one in ten was a violent crime.	DS	False	Share of violent offences among left-wing extremist crimes (2022).	Bundesamt für Verfassungsschutz (2023). Verfassungsschutzbericht 2022.	Task-Right	Bundeszentrale für politische Bildung (2023). Linksextremismus.

No.	Item Text: German Original	Item Text: English Translation	ID	Truth Value of Claim	Empirical Evidence	Empirical Source	Ideological Congruence of Correct Response	Ideological Framing Source
26	Die Anzahl der vom Verfassungsschutz erfassten linksextremistischen Personen nimmt in Deutschland seit über fünf Jahren stetig zu.	The amount of left-wing extremist individuals in Germany, recorded by the German domestic intelligence service, has been steadily increasing for more than five years.	DS	True	Number of individuals classified as left-wing extremist (time series).	Bundesamt für Verfassungsschutz (2023). Verfassungsschutzbericht 2022.	Task-Right	Hanns-Seidel-Stiftung (2023). Sicherheitspolitische Analysen.
27*	In Zeiten vorübergehender Grenzkontrollen Anfang des Jahres 2024 war ein Rückgang gestellter Asylanträge zu verzeichnen.	During periods of temporary border controls at the beginning of 2024, a decline in asylum applications was observed.	DS	True	Monthly asylum applications during temporary border controls (2024).	Bundesamt für Migration und Flüchtlinge (2024). Asylgeschäftsstatistik.	Task-Right	European Council on Foreign Relations (2023). Migration policy debates.
28	Die Anzahl der in Deutschland lebenden Menschen ohne deutschen Pass hat in den letzten fünf Jahren abgenommen.	The amount of people living in Germany without German citizenship has decreased during the last five years.	DS	False	Non-German resident population in Germany (time series).	Integrationsbeauftragte der Bundesregierung (2024). 14. Integrationsbericht.	Task-Right	DeZIM Institut (2024). Integrationsbericht 2024.
29	Die Anzahl der Asylanträge ist heute geringer als vor zehn Jahren.	There are less asylum applications today than there were ten years ago.	DS	False	Annual asylum applications (2014 vs recent years).	Statistisches Bundesamt (2024). Asylstatistik.	Task-Right	European Stability Initiative (2023). Asylum policy analysis.
30	Heute gehören mehr als doppelt so viele Personen der salafistischen Szene an als vor zehn Jahren.	Today, more than twice as many people belong to the Salafist scene than ten years ago.	DS	True	Number of individuals belonging to the Salafist scene (2012–2022 time series).	Bundesamt für Verfassungsschutz (2023). Verfassungsschutzbericht 2022.	Task-Right	Counter Extremism Project (2023). Germany extremism brief.

Notes. Items marked with an asterisk (*) were part of the initial candidate pool in Study 1 but were not retained in the final 20-item instrument following psychometric evaluation; ID = issue-domain; CC = climate change/renewable energy; DS = domestic security/migration; Ideological framing sources reference typical, issue-specific interpretations, grounding a claim's classification as Task-Left or Task-Right in observable public communication. These provide exemplary positions, not exhaustive or exclusive positions. Empirical sources were retrieved in March 2024; sources for ideological framing were retrieved in February 2025. Full references and corresponding links for all empirical and ideological framing sources are provided in the bibliography below.

Item Source Bibliography

ADAC. (2022). Lebenszyklusanalyse von Pkw: Vergleich von Elektrofahrzeugen und Verbrennern. https://www.adac.de/rund-ums-fahrzeug/elektromobilitaet/info/lebenszyklus/

AfD-Fraktion im Deutschen Bundestag. (2019). Gegenwind für das E-Auto (Drucksache 19/12231). Deutscher Bundestag. https://dserver.bundestag.de/btd/19/122/1912231.pdf

Agora Energiewende. (2024). Die Energiewende in Deutschland: Stand der Dinge 2024. https://www.agora-energiewende.de/

Agora Verkehrswende. (2022). Klimabilanz von Elektroautos. https://www.agora-verkehrswende.de/

Alfred-Wegener-Institut & Universität Bremen. (2020). Meereisportal: Zeitreihen und Trends der arktischen Meereisausdehnung. https://meereisportal.de/

Amadeu Antonio Stiftung. (2023). Jahresreport Rechtsextremismus 2023. https://www.amadeu-antonio-stiftung.de/publikationen/jahresbericht-2023/

Bundesamt für Migration und Flüchtlinge. (2024). Asylgeschäftsstatistik 2024. https://www.bamf.de/DE/Themen/Statistik/Asylgeschaeftsstatistik/asylgeschaeftsstatistik-node.html

Bundesamt für Migration und Flüchtlinge. (2024). Bundesamt in Zahlen 2023: Asyl, Migration und Integration. https://www.bamf.de/SharedDocs/Anlagen/DE/Statistik/BundesamtinZahlen/bundesamt-in-zahlen-2023.html

Bundesamt für Verfassungsschutz. (2023). Verfassungsschutzbericht 2022. https://www.verfassungsschutz.de/SharedDocs/publikationen/DE/verfassungsschutzberichte/2023-06-20-verfassungsschutzbericht-2022.html

Bundeskriminalamt. (2023). Gewalt gegen Polizeivollzugsbeamtinnen und Polizeivollzugsbeamte: Bundeslagebild 2022. https://www.bka.de/

Bundeskriminalamt. (2024). Bundeslagebild Rauschgiftkriminalität 2023. https://www.bka.de/

Bundeskriminalamt. (2024). Polizeiliche Kriminalstatistik 2023. https://www.bka.de/DE/AktuelleInformationen/StatistikenLagebilder/PolizeilicheKriminalstatistik/PKS2023/pks2023_node.html

Bundesministerium des Innern und für Heimat. (2023). Migrationsbericht der Bundesregierung 2022. https://www.bmi.bund.de/

Bundesministerium für Wirtschaft und Klimaschutz. (2023). Energiedaten: Gesamtausgabe. https://www.bmwk.de/Redaktion/DE/Artikel/Energie/energiedaten-gesamtausgabe.html

Bundeszentrale für politische Bildung. (2023). Linksextremismus. https://www.bpb.de/themen/linksextremismus-dossier/

Counter Extremism Project. (2023). Germany: Extremism and counter-extremism. https://www.counterextremism.com/countries/germany

Dessecker, A. (2021). Vom Recht und seiner Realität: Strafvollzug in Deutschland. Aus Politik und Zeitgeschichte, 71(42–43), 10–16. https://www.bpb.de/shop/zeitschriften/apuz/strafrecht-2021/

Deutscher Bauernverband. (2019). Klimastrategie 2.0 der deutschen Landwirtschaft. https://www.bauernverband.de/fileadmin/user_upload/dbv/positionen/Klimastrategie_2.0_2._Auflage_Januar_2019.pdf

Deutsche Polizeigewerkschaft. (2023). Gewalt gegen Polizeibeamtinnen und Polizeibeamte. https://www.dpolg.de/

DeZIM-Institut. (2024). Integrationsbericht 2024. https://www.dezim-institut.de/

European Council on Foreign Relations. (2023). Europe's migration politics after 2015. https://ecfr.eu/

European Monitoring Centre for Drugs and Drug Addiction. (2023). European Drug Report 2023: Trends and Developments. https://www.emcdda.europa.eu/publications/european-drug-report/2023_en

European Stability Initiative. (2023). The new politics of asylum in Europe. https://www.esiweb.org/

Expertenrat für Klimafragen. (2023). Prüfbericht zur Berechnung der deutschen Treibhausgasemissionen für das Jahr 2022. https://expertenrat-klima.de/

Expertenrat für Klimafragen. (2023). Zweijahresgutachten 2023. https://expertenrat-klima.de/

Friedlingstein, P., O'Sullivan, M., Jones, M. W., Andrew, R. M., Bakker, D. C. E., Hauck, J., … Peters, G. P. (2023). Global carbon budget 2023. Earth System Science Data, 15(12), 5301–5369. https://doi.org/10.5194/essd-15-5301-2023

Friedrich-Naumann-Stiftung für die Freiheit. (2023). Energiepolitik nach dem Atomausstieg. https://www.freiheit.org/de/deutschland/energiepolitik

Germanwatch. (2021, October 28). G20-Staaten verantwortlich für rund 75 Prozent der globalen Emissionen [Press release]. https://www.germanwatch.org/de/84318

Global Carbon Atlas. (2024). CO_2 emissions per capita. https://globalcarbonatlas.org/

Greenpeace Deutschland. (n.d.). Klimagerechtigkeit: Wer trägt Verantwortung für die Klimakrise? https://www.greenpeace.de/klimaschutz/klimakrise/klimagerechtigkeit

Hanns-Seidel-Stiftung. (2023). Linksextremismus als Herausforderung der inneren Sicherheit. https://www.hss.de/

Häusler, A. (2019). Die AfD und der Klimawandel: Zwischen Leugnung und national-ökologischer Wende. Heinrich-Böll-Stiftung. https://www.boell.de/de/2019/07/17/die-afd-und-der-klimawandel

Integrationsbeauftragte der Bundesregierung. (2024). 14. Bericht der Beauftragten der Bundesregierung für Migration, Flüchtlinge und Integration. https://www.integrationsbeauftragte.de/

Intergovernmental Panel on Climate Change. (2023). AR6 synthesis report: Climate change 2023. https://www.ipcc.ch/report/ar6/syr/

Klimafakten. (2023). Warum die Arktis für die Klimadebatte so wichtig ist. https://www.klimafakten.de/meldung/warum-die-arktis-fuer-die-klimadebatte-so-wichtig-ist

Konrad-Adenauer-Stiftung. (2023). Migrationspolitische Debatten in Deutschland und Europa. https://www.kas.de/de/migrationspolitische-debatten

Mediendienst Integration. (2023). Faktencheck: Kriminalität und Herkunft. https://mediendienst-integration.de/artikel/kriminalitaet-und-herkunft.html

Mediendienst Integration. (2024). Begehen Ausländer öfter Straftaten als Deutsche? https://mediendienst-integration.de/kriminalitaet/auslaenderkriminalitaet/begehen-auslaender-oefter-straftaten-als-deutsche/

Sachverständigenrat für Integration und Migration. (2023). Jahresgutachten 2023: Rückkehrpolitik und Abschiebung im politischen Konfliktfeld. https://www.svr-migration.de/publikationen/jahresgutachten/

Statistisches Bundesamt. (2024). Asylstatistik. https://www.destatis.de/

Statistisches Bundesamt. (2024). Strafvollzugsstatistik. https://www.destatis.de/

Umweltbundesamt. (2022). National inventory report for the German greenhouse gas inventory 1990–2020. https://www.umweltbundesamt.de/

Umweltbundesamt. (2024). Treibhausgas-Emissionen in Deutschland seit 1990. https://www.umweltbundesamt.de/daten/klima/treibhausgas-emissionen-in-deutschland

Umweltbundesamt. (2024). Zeitreihen zur Entwicklung der erneuerbaren Energien in Deutschland. https://www.umweltbundesamt.de/

UNFCCC. (2023). Outcome of the first global stocktake. https://unfccc.int/process-and-meetings/the-paris-agreement/global-stocktake
