# Supplementary material for: Ideologically biased evaluation of evidence - A signal-detection approach to measure individual differences
Source: PLoS One. 2026 Sep 11;21(9):e0357011. doi: 10.1371/journal.pone.0357011 (PMC13568519; doi:10.1371/journal.pone.0357011)
Supplement: Supplementary file 3 [file pone.0357011.s003.pdf]

Table S3. Maintenance Framework for IBE Items.

No.	Empirical Evidence	Empirical Source	Update Frequency Empirical Source	Item type / Temporal Sensitivity	Invalidation Criterion	Verification interval
1	Germany's share of global CO_2 emissions relative to its share of global population (cross-sectional comparison).	Friedlingstein, P., et al. (2023). Global carbon budget 2023. Earth System Science Data.	Annual	Threshold / Moderate	Germany's emissions share declines below 1% relative to global population share.	Annual
3	Arctic summer sea ice extent comparison (1980 vs. present).	Alfred-Wegener-Institut & Universität Bremen (2020). Meereisportal trend curves.	Annual	Threshold / Low	Present ice coverage recovers to less than one-third in difference compared to 1980.	Every 3–5 years
4	G20 aggregate greenhouse-gas emissions relative to global total.	Intergovernmental Panel on Climate Change (2023). AR6 synthesis report.	Annual	Threshold / Low	G20 share crosses the one-third threshold.	Annual
5	Share of livestock-related emissions in Germany's agricultural GHG emissions.	Umweltbundesamt (2022). National inventory report for the German greenhouse gas inventory.	Annual	Threshold / Low	Livestock share deviates substantially from the ~5% benchmark.	Annual
6	Per-capita fossil CO_2 emissions comparison between Germany and China.	Global Carbon Atlas (2024).	Annual	Threshold / Moderate	Germany's per-capita emissions fall below China's level.	Annual
9	Nationality distribution of burglary suspects (PKS).	Bundeskriminalamt (2024). Polizeiliche Kriminalstatistik 2023.	Annual	Threshold / Low	German suspects no longer constitute the majority.	Annual
11	Right-wing extremist offences recorded in Germany (time series).	Bundesamt für Verfassungsschutz (2023). Verfassungsschutzbericht 2022.	Annual	Trend / Moderate	Ten-year trend reverses.	Annual
12	Overall protection rate of asylum applications in Germany.	Bundesamt für Migration und Flüchtlinge (2024). Bundesamt in Zahlen 2023.	Annual	Threshold / Moderate	Protection rate crosses the 80% threshold.	Annual
13	Executed deportations from Germany (2020–2023 time series).	Bundesministerium des Innern und für Heimat (2023). Migrationsbericht.	Annual	Trend / Moderate	Deportations cease increasing relative to 2020 baseline.	Annual
16	Global mean surface temperature change relative to pre-industrial baseline.	IPCC (2023). AR6 synthesis report.	Annual	Threshold / High	Global warming exceeds 1.5°C.	Annual
17	Electricity price trends before and after nuclear phase-out.	Bundesministerium für Wirtschaft und Klimaschutz (2023). Energy data portal.	Annual	Trend / Low	Prices no longer increase following nuclear phase-out.	Annual

No.	Empirical Evidence	Empirical Source	Update Frequency Empirical Source	Item type / Temporal Sensitivity	Invalidation Criterion	Verification interval
19	Total GHG emissions in Germany (time series).	Umweltbundesamt (2024). Treibhausgas-Emissionen Zeitreihe.	Annual	Trend / Low	Emissions show sustained increase instead of decline.	Annual
20	Production-phase energy demand comparison between EV and ICE vehicles.	ADAC (2022). Lebenszyklusanalyse-Tool.	Irregular	Threshold / Moderate	EV production energy no longer exceeds ICE production energy.	Annual
21	Share of renewable energy in Germany's gross final energy consumption (time series).	Umweltbundesamt (2024). Zeitreihen erneuerbare Energien.	Annual	Threshold / Low	Renewable share no longer reflects doubling within the ten-year window.	Annual
22	Share of non-German suspects among all suspects (PKS).	Bundeskriminalamt (2024). Polizeiliche Kriminalstatistik 2023.	Annual	Threshold / Low	Foreign suspects exceed one-third of offenders.	Annual
24	Violence against police officers in Germany (time series).	Bundeskriminalamt (2023). Gewalt gegen Polizeivollzugsbeamt*innen – Bundeslagebild.	Annual	Trend / Moderate	Long-term increase reverses.	Annual
26	Number of individuals classified as left-wing extremist (time series).	Bundesamt für Verfassungsschutz (2023). Verfassungsschutzbericht 2022.	Annual	Trend / Moderate	Five-year trend reverses.	Annual
28	Non-German resident population in Germany (time series).	Integrationsbeauftragte der Bundesregierung (2024). 14. Integrationsbericht.	Annual	Trend / Moderate	Five-year trend shows decline.	Annual
29	Annual asylum applications (2014 vs recent years).	Statistisches Bundesamt (2024). Asylstatistik.	Annual	Threshold / High	Current applications exceed the level ten years earlier.	Annual
30	Number of individuals belonging to the Salafist scene (2012–2022 time series).	Bundesamt für Verfassungsschutz (2023). Verfassungsschutzbericht 2022.	Annual	Threshold / Moderate	Population no longer exceeds twice the level ten years earlier.	Annual

Notes. Threshold items assess whether an empirical value crosses a specified benchmark, whereas trend items assess the direction or persistence of a temporal development. To support long-term usability, each item was assigned a maintenance protocol specifying the update frequency of the underlying statistic, item type, temporal sensitivity of the empirical claim, invalidation criterion, and verification interval. The invalidation criterion defines the empirical condition requiring revision of the item wording (a threshold being surpassed or a trend reversing), while the verification interval specifies how frequently the underlying statistic should be checked for updates. This procedure allows item wording to remain stable while ensuring that the empirical benchmarks underlying item correctness remain accurate over time.
