# Supplementary material for: Ideologically biased evaluation of evidence - A signal-detection approach to measure individual differences
Source: PLoS One. 2026 Sep 11;21(9):e0357011. doi: 10.1371/journal.pone.0357011 (PMC13568519; doi:10.1371/journal.pone.0357011)
Supplement: Supplementary file 4 [file pone.0357011.s004.pdf]

Table S4. Logistic Regression Predicting Candidate Item Responses from Political Knowledge and Political Orientation (Study 1).

No.	Ideol. Congruence Correct Response	Issue-Domain	Truth Value of Claim	*M*	Odds Ratios 95% CI (LL, UL)	
					HIP	POL
1	Task-Left	CC	False	.51	2.724 (1.045, 7.224)	0.859 (0.774, 0.949)
2*	Task-Left	CC	True	.67	1.812 (0.661, 4.942)	0.838 (0.748, 0.933)
3	Task-Left	CC	True	.89	5.173 (1.236, 20.981)	0.869 (0.736, 1.021)
4	Task-Left	CC	False	.60	12.117 (4.48, 33.977)	0.934 (0.841, 1.036)
5	Task-Left	CC	True	.73	9.055 (3.182, 26.31)	0.854 (0.757, 0.959)
6	Task-Left	CC	False	.26	6.007 (1.866, 20.747)	0.867 (0.774, 0.97)
7*	Task-Left	DS	True	.16	0.848 (0.243, 3.132)	0.964 (0.845, 1.101)
8*	Task-Left	DS	True	.27	1.188 (0.411, 3.541)	0.92 (0.824, 1.027)
9	Task-Left	DS	True	.33	2.696 (0.933, 8.074)	0.737 (0.656, 0.823)
10*	Task-Left	DS	False	.86	36.507 (10.152, 137.115)	0.916 (0.787, 1.061)
11	Task-Left	DS	True	.86	1.873 (0.487, 6.882)	0.782 (0.669, 0.907)
12	Task-Left	DS	False	.48	6.611 (2.464, 18.396)	0.891 (0.804, 0.985)
13	Task-Left	DS	True	.41	2.522 (0.944, 6.91)	0.851 (0.767, 0.942)
14*	Task-Left	DS	True	.37	2.787 (1.015, 7.901)	0.831 (0.747, 0.921)
15*	Task-Left	DS	False	.21	0.097 (0.031, 0.295)	0.901 (0.798, 1.017)
16	Task-Right	CC	True	.26	0.608 (0.212, 1.771)	1.166 (1.04, 1.312)
17	Task-Right	CC	False	.78	0.154 (0.04, 0.551)	1.356 (1.201, 1.537)
18*	Task-Right	CC	True	.37	4.263 (1.545, 12.242)	1.095 (0.988, 1.216)
19	Task-Right	CC	False	.35	3.074 (1.113, 8.815)	1.035 (0.934, 1.148)
20	Task-Right	CC	True	.79	5.619 (1.828, 17.309)	1.144 (1.014, 1.292)
21	Task-Right	CC	True	.79	4.449 (1.45, 13.57)	0.942 (0.832, 1.064)

No.	Ideol. Congruence Correct Response	Issue-Domain	Truth Value of Claim	*M*	Odds Ratios 95% CI (LL, UL)	
					HIP	POL
22	Task-Right	DS	False	.47	0.754<br>(0.285, 1.988)	1.272<br>(1.146, 1.421)
23*	Task-Right	DS	True	.69	0.424<br>(0.144, 1.204)	1.091<br>(0.982, 1.214)
24	Task-Right	DS	True	.95	23.479<br>(3.449, 163.669)	1.295<br>(1.047, 1.598)
25*	Task-Right	DS	False	.61	1.444<br>(0.543, 3.822)	1.172<br>(1.059, 1.301)
26	Task-Right	DS	True	.76	2.551<br>(0.851, 7.56)	1.184<br>(1.055, 1.33)
27*	Task-Right	DS	True	.57	1.707<br>(0.66, 4.428)	1.076<br>(0.975, 1.189)
28	Task-Right	DS	False	.81	7.279<br>(2.308, 23.079)	1.104<br>(0.974, 1.252)
29	Task-Right	DS	False	.87	1.499<br>(0.359, 5.874)	1.222<br>(1.059, 1.41)
30	Task-Right	DS	True	.67	2.357<br>(0.865, 6.414)	1.172<br>(1.056, 1.305)

Note. $N = 446$; Items marked with an asterisk (*) were part of the initial candidate pool in Study 1 but were not retained in the final 20-item instrument following psychometric evaluation. Logistic regression models were estimated to examine the probability of a correct response as a function of political knowledge and political orientation. CC = issue-domain climate change/renewable energy; DS = issue-domain domestic security/migration; HIP = Hohenheimer Inventory for Political Knowledge (Trepte et al., 2017); POL = Political Orientation (see Study 1 methods), *M* = proportion of correct responses.
