# Supplementary material for: Ideologically biased evaluation of evidence - A signal-detection approach to measure individual differences
Source: PLoS One. 2026 Sep 11;21(9):e0357011. doi: 10.1371/journal.pone.0357011 (PMC13568519; doi:10.1371/journal.pone.0357011)
Supplement: Supplementary file 5 [file pone.0357011.s005.pdf]

Table S5. Split-Half Reliability for Discrimination Sensitivity and Ideological Bias Expression.

Parameter	Study 1		Study 2	
	uncorrected	Spearman-Brown	uncorrected	Spearman-Brown
d′	.13	.23	.14	.24
b′	.49	.65	.44	.61

Note. N_{Study1} = 446; N_{Study2} = 1359; *d′* = discrimination sensitivity, *b′* = ideological bias expression.
