# Supplementary material for: Ideologically biased evaluation of evidence - A signal-detection approach to measure individual differences
Source: PLoS One. 2026 Sep 11;21(9):e0357011. doi: 10.1371/journal.pone.0357011 (PMC13568519; doi:10.1371/journal.pone.0357011)
Supplement: Supplementary file 6 [file pone.0357011.s006.pdf]

Table S6. Task-Specific Difficulty and Discrimination Distributions by Domain.

Ideol. Congruence of Correct Response	Issue-Domain	Difficulty		Discrimination	
		P_T	*SD*	*M*	*SD*
Task-Left	CC	.63	.23	.31	.06
Task-Left	DS	.52	.23	.25	.14
Task-Right	CC	.55	.28	.12	.10
Task-Right	DS	.75	.17	.23	.06

Note. $N_{Study\ 1}$ = 446; CC = issue domain climate change/renewable energy; DS = issue domain domestic security/migration; P_T = mean probability of correct responses across items within the task.
