# Supplementary material for: Ideologically biased evaluation of evidence - A signal-detection approach to measure individual differences
Source: PLoS One. 2026 Sep 11;21(9):e0357011. doi: 10.1371/journal.pone.0357011 (PMC13568519; doi:10.1371/journal.pone.0357011)
Supplement: Supplementary file 7 [file pone.0357011.s007.pdf]

Table S7. Summary of Multilevel Logistic Regression Predicting Probability of Correct Responses.

Predictor	*b*	*SE*	95% CI [LL, UL]	*p*
Intercept	0.46	0.35	[-0.23, 1.14]	0.192
Ideology	-0.28	0.04	[-0.35, -0.21]	< .001
Congruence (Right)	0.46	0.50	[-0.51, 1.44]	0.350
Ideology × Congruence (Right)	0.60	0.05	[0.50, 0.70]	< .001

Random Effects	*Variance*	*SD*
Item	1.22	1.10
Respondent	0.05	0.23

Notes. The analysis was based on 8,920 respondent–item observations from 446 respondents and 20 items. Model fit: AIC = 9781.60; BIC = 9824.20; log-likelihood = -4884.80. Entries are unstandardized log-odds coefficients from a multilevel logistic regression model. Congruence was dummy coded (0 = left-congruent, 1 = right-congruent), and ideology was standardized ($M = 0$, $SD = 1$). Random intercepts were included for respondents and items. CI = confidence interval.
