# Supplementary material for: Ideologically biased evaluation of evidence - A signal-detection approach to measure individual differences
Source: PLoS One. 2026 Sep 11;21(9):e0357011. doi: 10.1371/journal.pone.0357011 (PMC13568519; doi:10.1371/journal.pone.0357011)
Supplement: Supplementary file 8 [file pone.0357011.s008.pdf]

Table S8. Summary of Reversed Hierarchical Regression Models for predicting Ideological Bias Expression (b').

Predictor	Model 1<br>β<br>95% CI [LL, UL]	Model 2b<br>β<br>95% CI [LL, UL]	Model 3b<br>β<br>95% CI [LL, UL]
Age	.065*<br>[0.000, 0.007]	.024 (n.s.)<br>[−0.001, 0.004]	.033 (n.s.)<br>[−0.001, 0.004]
Sex	.011 (n.s.)<br>[−0.080, 0.113]	.065**<br>[0.029, 0.174]	.062**<br>[0.026, 0.169]
Party ID (L–R)	—	—	.183*<br>[0.072, 0.139]
Sustainability	—	- .336*<br>[−0.259, −0.192]	- .286***<br>[−0.227, −0.157]
Security	—	- .034 (n.s.)<br>[−0.110, 0.025]	- .035 (n.s.)<br>[−0.111, 0.022]
Tradition	—	.200*<br>[0.111, 0.201]	.174***<br>[0.091, 0.180]
Anti-Immigration	—	.371*<br>[0.201, 0.278]	.299***<br>[0.153, 0.234]
Solidarity	—	- .021 (n.s.)<br>[−0.058, 0.024]	- .006 (n.s.)<br>[−0.045, 0.035]
Model Fit	$R^2 = .004$	$R^2 = .449$	$R^2 = .469$
Fit Change	—	$\Delta R^2 = .444$<br>$F(5, 997) = 115.90$***	$\Delta R^2 = .020$<br>$F(1, 996) = 37.45$***

Note. N = 1005 (listwise after excluding “Other/None" and "BSW”); β = standardized regression weight. CI = 95% confidence interval for B. Significance codes: n.s. = not significant. . $p < .10$, * $p < .05$, ** $p < .01$, *** $p < .001$.
