# Supplementary material for: Ideologically biased evaluation of evidence - A signal-detection approach to measure individual differences
Source: PLoS One. 2026 Sep 11;21(9):e0357011. doi: 10.1371/journal.pone.0357011 (PMC13568519; doi:10.1371/journal.pone.0357011)
Supplement: Supplementary file 9 [file pone.0357011.s009.pdf]

Table S9. Mapping of Political Party Ideological Positions to Coding Categories Based on MARPOR RILE and CHES Left–Right Expert Scores.

Political Party	Party Manifesto Latent Ideology Variable (rile) (-100 to +100)	CHES Cultural Issue Dimension Left–Right Expert Rating (0–10)	Coding Category	Code
DIE LINKE	-36.17	2.14	Far Left	-2
Bündnis 90/Die Grünen	-21.04	1.64	Moderate Left	-1
SPD	-24.67	3.59	Moderate Left	-1
CDU/CSU	3.49 (CDU)<br>5.00 (CSU)	6.23 (CDU)<br>7.56 (CSU)	Moderate Right	1
FDP	0.27	3.86	Moderate Right	1
AfD	26.00	8.55	Far Right	2

Notes. Coding categories were derived from baseline party positions as of 2021, based on the Manifesto Project's Latent Ideology Variable (rile); scale: -100 = most left-wing, +100 = most right-wing) and the Chapel Hill Expert Survey (CHES) left–right expert scale (0 = extreme left, 10 = extreme right).
