# Supplementary material for: Ideologically biased evaluation of evidence - A signal-detection approach to measure individual differences
Source: PLoS One. 2026 Sep 11;21(9):e0357011. doi: 10.1371/journal.pone.0357011 (PMC13568519; doi:10.1371/journal.pone.0357011)

Figure S1. Interaction between Ideology and Item Congruence on the Probability of Correct Responses.

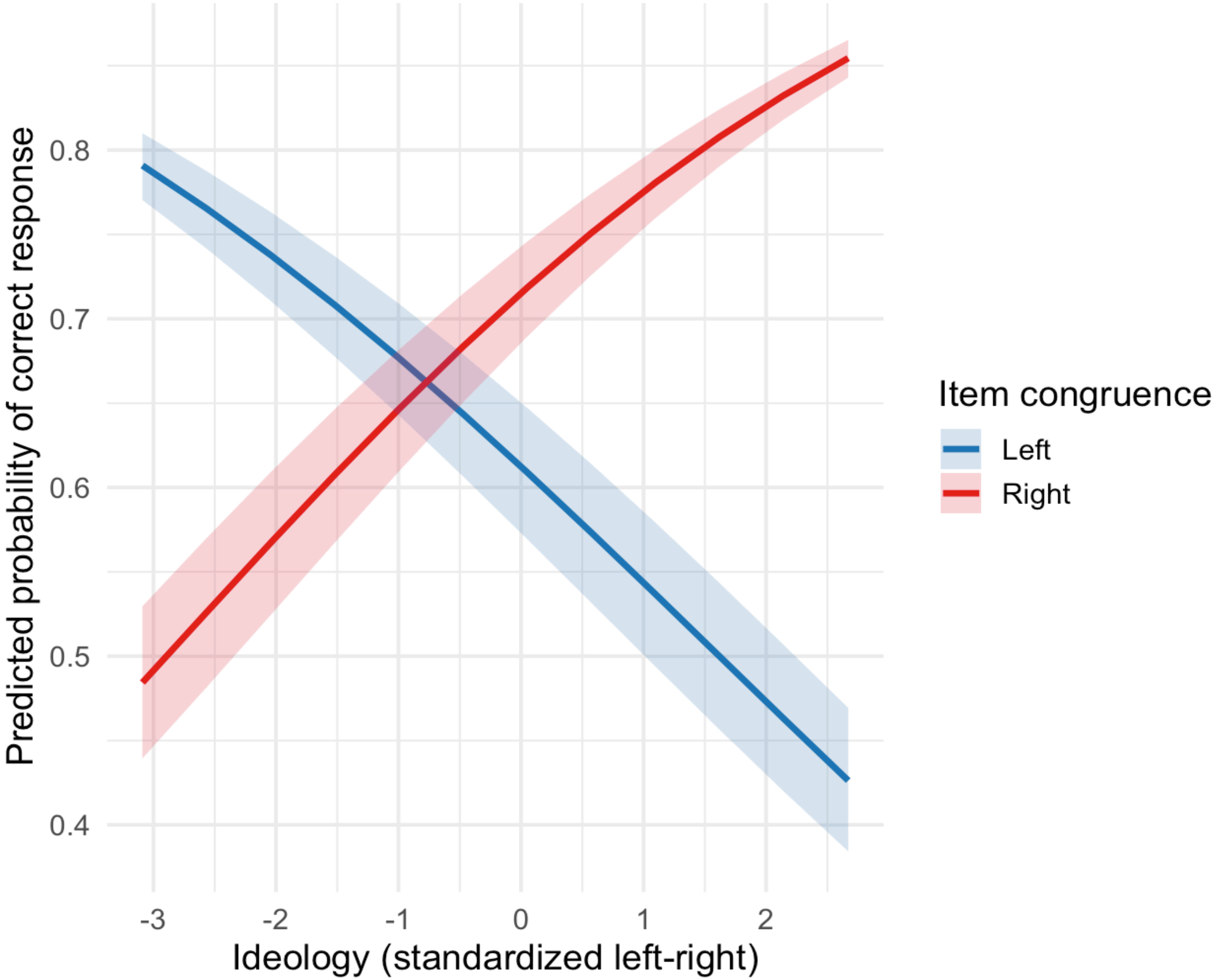

Supplement: Supplementary file 10 [file pone.0357011.s010.pdf]
